# Comparative effectiveness of neuromuscular electrical stimulation-based combination interventions for post-stroke lower limb rehabilitation: a systematic review and network meta-analysis

**DOI:** 10.1186/s12984-026-02009-3

**Published:** 2026-05-16

**Authors:** Kaiqi Zheng, Yuan Yang, Pengcheng Liu, Weidong Liang, Xiaokun Gong, Tiangeng Li, Liang Guo

**Affiliations:** 1 School of Physical Education and Sports Science, South China Normal University, No. 378, Outer Ring West Road, Guangzhou University Town, Panyu District, Guangzhou, 510000 Guangdong China; 2 Shuangliu District Shuanghua Primary School, Chengdu, 610200 Sichuan China

**Keywords:** Stroke, Neuromuscular electrical stimulation, Balance ability, Network meta-analysis, Systematic review

## Abstract

**Objective:**

This study aimed to comparatively evaluate the efficacy of different neuromuscular electrical stimulation (NMES)-based combination protocols for improving lower limb dysfunction in patients with stroke, in order to provide evidence-based support for clinical rehabilitation practice.

**Methods:**

Randomized controlled trials (RCTs) investigating NMES-based combination interventions for lower limb dysfunction in patients with stroke published up to September 2, 2025 were systematically identified through searches of PubMed, Embase, the Cochrane Library, and Web of Science. The risk of bias of eligible studies was evaluated using the Cochrane Risk of Bias 2.0 (ROB 2.0) tool. Network meta-analysis (NMA) and subgroup analyses were conducted using R software (version 4.5.1) and Stata (version 15.0).

**Results:**

In total, 37 RCTs were incorporated into the present NMA, involving 1,764 patients with stroke and encompassing nine distinct NMES-based combination intervention protocols. Regarding balance ability, core stability training (CST) combined with NMES (SMD = 0.99, 95% CrI: 0.33–1.67), gait training (GT) combined with NMES (SMD = 0.83, 95% CrI: 0.35–1.27), and NMES alone (SMD = 0.71, 95% CrI: 0.21–1.22) demonstrated significant therapeutic effects. CST+NMES (SUCRA = 76.02%) ranked highest among the NMES-based combined interventions for balance ability in patients with stroke. With respect to walking ability, no statistically significant differences were observed among the NMES-based intervention comparisons. Nevertheless, cycling exercise (CE) combined with NMES (SUCRA = 77.46%) received the highest ranking probability for walking ability, although this exploratory finding should be interpreted with caution given the absence of statistically significant differences among interventions and the lack of closed loops in the network. In terms of lower limb functional mobility, augmented reality technology (ART) combined with GT and NMES (SMD = 1.38, 95% CrI: 0.25–2.52), GT+NMES (SMD = 0.85, 95% CrI: 0.39–1.36), and NMES alone (SMD = 0.55, 95% CrI: 0.06–1.05) showed significant improvements. ART+GT+NMES (SUCRA = 84.23%) ranked highest for lower limb functional mobility. The results of the two-dimensional cluster ranking indicated that GT+NMES (SUCRA = 65.22%/49.29%/62.22%) demonstrated relatively stable ranking performance across multiple domains of lower limb dysfunction in patients with stroke. Subgroup analyses further indicated that CST+NMES appeared to be more effective for patients in the acute phase, GT+NMES for those in the subacute phase, and ART+GT+NMES for patients in the chronic phase. These subgroup-level rankings were based on limited evidence and should be considered exploratory.

**Conclusions:**

NMES-based combination strategies have demonstrated beneficial effects on the recovery of lower limb function among individuals after stroke. Based on the results of this NMA, GT+NMES demonstrated relatively stable ranking performance across multiple outcomes, including balance, walking ability, and lower limb functional mobility, suggesting that it warrants further investigation as a combined intervention for clinical application. In addition, patients at different stages of stroke showed variable responses to NMES-based combined interventions, highlighting the importance of stage-specific and individualized rehabilitation strategies.

*Trial registration:* PROSPERO CRD420251229835.

**Supplementary Information:**

The online version contains supplementary material available at 10.1186/s12984-026-02009-3.

## Introduction

Stroke is a prevalent neurological condition resulting from either cerebral ischemia or intracranial hemorrhage and is characterized by high rates of morbidity, mortality, and recurrence worldwide [1, 2]. Impairments of lower limb function commonly occur following stroke and are typically reflected by gait disturbances, reduced muscle strength, and deficits in postural control, leading to considerable limitations in daily activities and overall quality of life [3, 4]. Consequently, improving lower extremity motor function represents one of the central objectives in post-stroke rehabilitation practice [5].

Neuromuscular electrical stimulation (NMES) is widely applied in post-stroke rehabilitation and has demonstrated beneficial effects on the recovery of lower limb motor dysfunction. This modality delivers controlled low-frequency electrical impulses to peripheral motor nerves or target muscles through surface or implanted electrodes, eliciting involuntary muscle contractions to facilitate functional improvement [6]. At present, NMES is commonly incorporated into rehabilitation programs for individuals with stroke-related lower limb dysfunction [7, 8]. Evidence from clinical studies indicates that NMES provides additional benefits beyond conventional rehabilitation approaches. For example, Almutairi et al. [9] observed superior improvements in walking performance among stroke patients receiving NMES compared with those undergoing standard rehabilitation. Similarly, Tan et al. [10] reported significant enhancements in lower extremity functional mobility and balance following NMES intervention. Consistent with these findings, two meta-analyses have further confirmed the overall effectiveness of NMES for improving lower limb function in patients with stroke [7, 8].

An expanding body of clinical evidence suggests that combining NMES with other rehabilitation approaches may further enhance recovery of lower limb function after stroke. For instance, Park et al. [11] observed greater improvements in lower limb functional mobility with the addition of motor imagery training to NMES compared with conventional rehabilitation alone. Similarly, Ko et al. [12] demonstrated that pairing NMES with core stability training resulted in superior balance improvements relative to both standard rehabilitation and NMES as a standalone intervention. Collectively, these findings highlight the potential value of NMES-based combination strategies in broadening the scope and effectiveness of post-stroke lower limb rehabilitation. However, despite the growing interest in combined NMES interventions, much of the existing literature has primarily examined the effects or underlying mechanisms of individual NMES protocols. Direct comparative evidence across different NMES-based combination strategies remains scarce, and a comprehensive synthesis identifying which combinations yield more favorable rehabilitation outcomes is still lacking.

By integrating comparative evidence across NMES-based combination approaches, this network meta-analysis (NMA) aims to clarify how different intervention strategies perform in improving lower limb function after stroke, thereby supporting more informed rehabilitation decision-making.

## Methods

Reporting of this NMA adhered to the PRISMA-NMA 2020 framework to ensure transparency and methodological rigor [13]. Prior to study initiation, the review protocol was prospectively recorded in the PROSPERO database (CRD420251229835).

### Search strategy

Literature retrieval was undertaken using four major electronic databases—PubMed, Embase, the Cochrane Library, and Web of Science—to identify relevant studies published up to September 2, 2025. Tailored search strategies were applied for each database to account for differences in indexing systems and search capabilities. Detailed search terms and strategies are provided in Supplementary Table 1.

### Inclusion and exclusion criteria

To ensure a clearly defined research question, minimize systematic bias, and maintain clinical generalizability, detailed and stringent inclusion and exclusion criteria were established for this NMA.

Inclusion criteria were as follows: (1) Participants: Stroke patients diagnosed by CT or MRI, aged ≥ 18 years, with no restrictions on sex, country, ethnicity, and disease duration. (2) Interventions in the experimental group: The experimental interventions included NMES alone or NMES combined with other rehabilitation modalities. In this NMA, intervention nodes were coded based on the actual treatment structure of each trial rather than the terminology used by the original authors. Accordingly, interventions described as FES were not automatically treated as a separate node. If electrical stimulation was delivered as part of a combined treatment program together with a clearly defined training modality (e.g., gait training or cycling exercise), the intervention was classified under the corresponding NMES-based combination node (e.g., gait training combined with NMES). In contrast, if electrical stimulation was delivered as a standalone intervention without being combined with an actual training modality, it was classified as NMES, even if the original study described it as FES or used function-oriented stimulation patterns. For mixed or insufficiently reported protocols, classification was based on the original intervention description, and a conservative coding strategy was applied by assigning the study to the closest structural NMES node rather than treating FES as an independent node. (3) Interventions in the control group: The control group received conventional rehabilitation (C), such as routine physical therapy or standard rehabilitation care. Both the experimental and control groups received conventional rehabilitation as background therapy. The experimental intervention (NMES alone or NMES combined with other modalities) was delivered as an add-on to this shared background. (4) Outcomes: Outcome measures included the Berg Balance Scale (BBS), Postural Assessment Scale for Stroke Patients (PASS), Fugl-Meyer Assessment–Lower Extremity (FMA-LE), Timed Up and Go Test (TUGT), Brunnstrom Stages–Lower Extremity (BS-LE), Functional Ambulation Category Scale (FAC), and 10-Meter Walking Test (10MWT). Among these, BBS and PASS primarily assessed balance ability, BS-LE, FMA-LE, and TUGT primarily assessed lower limb functional mobility, and FAC and 10MWT primarily assessed walking ability. For most outcome measures (BBS, PASS, FMA-LE, BS-LE, FAC), higher scores reflect better function. For the TUGT and 10MWT, lower values indicate superior performance. The direction of all effect estimates was aligned prior to analysis so that positive values uniformly indicated functional improvement. (5) Study design: Eligibility was restricted to peer-reviewed randomized controlled trials (RCTs) that provided detailed outcome data.

Exclusion criteria were as follows: (1) Studies that were not peer-reviewed or publicly published. (2) Duplicate publications based on the same RCT. (3) Studies that did not report any of the aforementioned outcome measures. (4) Non-English language publications. This language restriction was applied to ensure the accuracy of data extraction, risk of bias assessment, and methodological quality evaluation, as reliable interpretation of full-text articles required proficiency in the publication language. While this is a common practice in systematic reviews, it may have excluded potentially relevant studies, particularly those published in Chinese, given the substantial volume of stroke rehabilitation research conducted in China. (5) Conference abstracts.

### Study selection and data extraction

Study identification and selection were carried out independently by two reviewers (Kaiqi Zheng and Weidong Liang). All records retrieved from the four electronic databases were compiled using EndNote X9, where duplicate citations were identified and removed electronically. Before initiating the formal screening process, the reviewers established unified eligibility criteria through discussion to ensure consistency in study selection. Any disagreements encountered during title/abstract or full-text screening were resolved through consultation with a third senior investigator (Liang Guo), who provided the final decision. Data extraction was subsequently performed using predesigned Excel data sheets by two reviewers. In this NMA, data extracted from the original studies were entered into an Excel spreadsheet. The extracted information mainly included the first author, publication year, mean age of participants in the experimental and control groups, sample size of the experimental and control groups, intervention protocols in the experimental and control groups, outcome measures, disease duration of participants in the experimental and control groups, NMES intervention period and frequency, stimulation target, application mode, stimulation intensity and frequency, pulse width, duty cycle, trigger mode, as well as detailed descriptions of the interventions implemented in both the experimental and control groups.

### Risk of bias assessment

The methodological rigor of the included randomized controlled trials was examined using the Cochrane Risk of Bias 2.0 (ROB 2.0) framework. To promote consistency in judgment, two reviewers (Kaiqi Zheng and Weidong Liang) completed standardized training and conducted calibration exercises on sample studies before undertaking the formal assessments. Each eligible study was subsequently evaluated independently, after which the reviewers compared their assessments. Any discrepancies were resolved through consultation with a third senior investigator (Liang Guo), who made the final determination. Risk of bias judgments were generated across five core domains encompassing the randomization process, adherence to intended interventions, completeness of outcome data, outcome assessment, and reporting practices. Domain-level judgments were informed by the corresponding signaling questions and categorized to reflect varying levels of potential bias. Overall risk of bias classifications were derived by synthesizing the pattern of judgments across domains, with studies exhibiting consistently favorable assessments considered to have lower risk, and those demonstrating substantial or cumulative concerns regarded as having higher risk of bias.

### Data analysis

Multiple software platforms were used in this study, each serving a distinct analytical role. Bayesian NMA was performed in R (version 4.5.1) using the gemtc package (version 1.1-0), with Markov chain Monte Carlo (MCMC) sampling conducted via JAGS (version 4.3.1) through the rjags package (version 4-17). The gemtc package facilitated consistency and inconsistency model fitting, node-splitting analyses, treatment ranking based on the surface under the cumulative ranking curve (SUCRA), and league table generation. The coda package (version 0.19-4.1) was used for convergence diagnostics, including trace plot inspection and the Gelman-Rubin statistic. Stata (version 15.0) was used for network plot construction, funnel plot generation, and cumulative ranking probability visualization. Continuous outcome measures were summarized as standardized mean differences (SMD) accompanied by 95% credible intervals (CrI). Within the Bayesian framework, treatment effects were considered statistically meaningful when the corresponding 95% CrI did not include zero. For subgroup comparisons involving only two interventions, pairwise meta-analysis was conducted using Review Manager (version 5.4), and statistical significance was determined using the conventional threshold of *p* < 0.05 with 95% confidence intervals (CI). Because different outcome measures were pooled within each functional domain, the direction of effect was standardized prior to analysis. For most included measures (BBS, PASS, FMA-LE, BS-LE, FAC), higher scores indicate better function, and change scores were calculated as post-intervention minus baseline values. For the TUGT and 10MWT, in which lower values indicate better performance, change scores were calculated as baseline minus post-intervention values. This ensured that positive SMD values consistently represented favorable treatment effects across all outcomes. All analyses were implemented under a random-effects consistency framework. Vague prior distributions were used for all model parameters following the default settings of the gemtc package. Relative treatment effects were assigned normal priors centered at zero with a large variance scaled to the observed data range. The between-study heterogeneity standard deviation (τ) was assigned a uniform prior bounded between zero and an upper limit derived from the data scale. These specifications represent standard non-informative priors for Bayesian NMA, designed to let the observed data dominate the posterior estimates. Between-study heterogeneity was quantified using the posterior distribution of τ, with its median and 95% CrI reported for each outcome. For network structures containing closed loops, node-splitting methods were applied to evaluate potential local inconsistencies. The consistency model was fitted using 20,000 burn-in iterations followed by 50,000 sampling iterations. Model convergence was assessed by visual inspection of trace plots and the Gelman–Rubin diagnostic, with all parameters demonstrating satisfactory convergence. Model fit was assessed by comparing the deviance information criterion (DIC) between consistency and inconsistency models, with a DIC difference of less than 5 indicating no meaningful inconsistency and adequate agreement between direct and indirect evidence. Treatment ranking was determined using the SUCRA, where higher values reflected a greater probability of being among the top-ranked interventions. It should be noted that SUCRA values represent ranking probabilities derived from the posterior distribution of treatment effects and do not directly indicate statistically significant differences between interventions. Therefore, SUCRA rankings were interpreted in conjunction with the corresponding effect estimates and credible intervals, and were not used as the sole basis for determining intervention superiority. To further explore interventions demonstrating favorable performance across multiple functional outcomes, a two-dimensional cluster ranking based on SUCRA values was conducted, with interventions consistently achieving higher rankings considered potential optimal therapeutic strategies. Publication bias for each outcome was evaluated using funnel plots. Subgroup analyses were conducted to explore the optimal NMES-based combined interventions for stroke patients at different stages post-onset. To assess the robustness of the main findings, a sensitivity analysis was performed by excluding studies rated as high overall risk of bias under the ROB 2.0 framework. Three studies (Kim 2012, Jovana 2009, and Salisbury 2013) were identified as high risk and were removed from the relevant outcome networks. The Bayesian NMA was then re-run using the same model specifications, and the resulting SUCRA rankings were compared with those from the main analysis to evaluate the stability of the treatment rankings. The confidence in the evidence for each pairwise comparison within the network was evaluated using the CINeMA (Confidence in Network Meta-Analysis) framework, which assesses six domains: within-study bias, reporting bias, indirectness, imprecision, heterogeneity, and incoherence. Each comparison was assigned a confidence rating of high, moderate, low, or very low based on the pattern of concerns identified across these domains. The CINeMA assessment was performed using the CINeMA web application (https://cinema.ispm.unibe.ch/).

## Results

### Search results

**Fig. 1:**
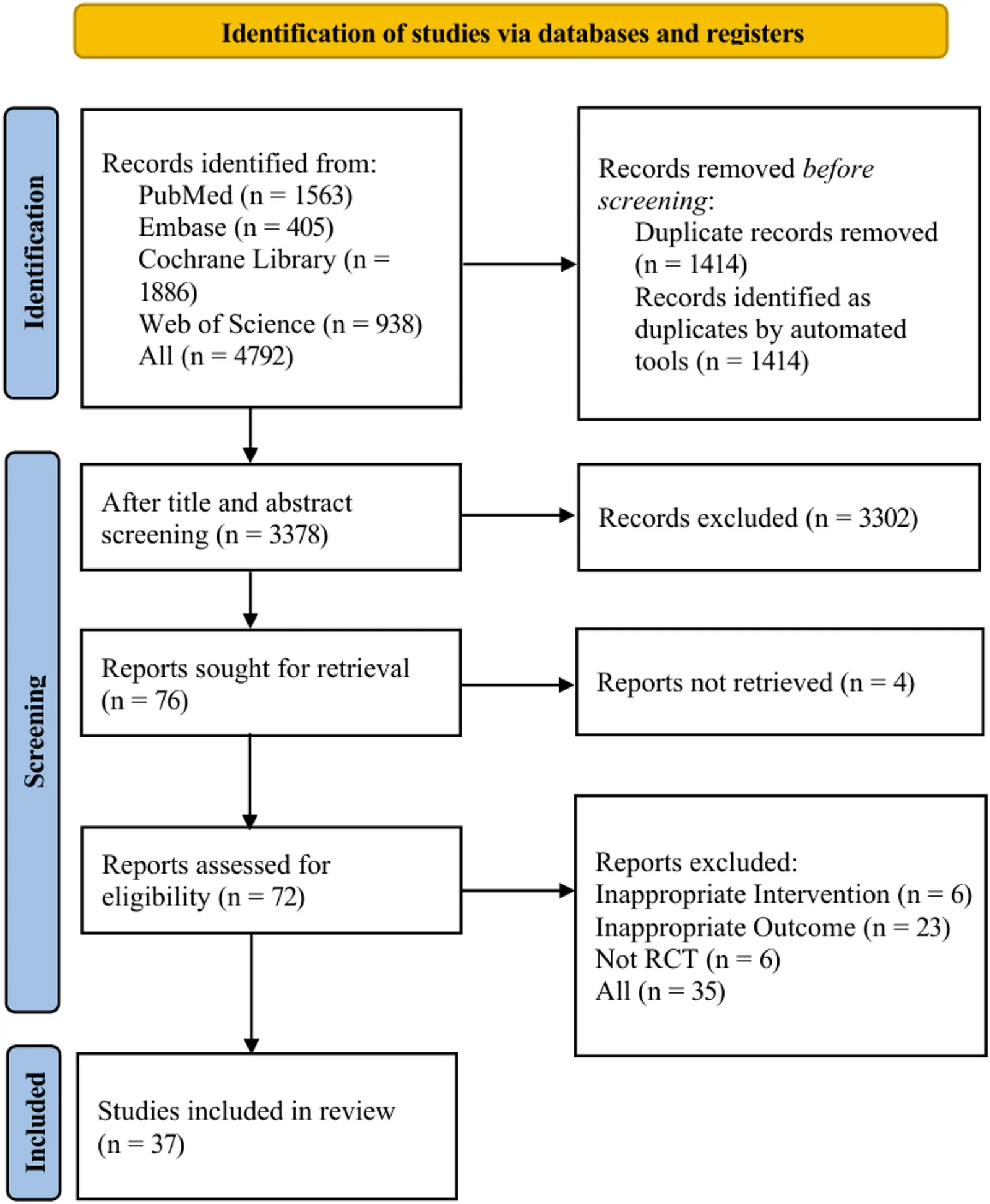
Flow chart of literature screening

The database searches yielded 4,792 records in total, as detailed in Supplementary Table 1, including 1,563 records from PubMed, 1,886 from the Cochrane Library, 405 from Embase, and 938 from Web of Science. After removing 1,414 duplicate records using the built-in deduplication function of EndNote X9, 3,378 records remained. Following title and abstract screening, 3,302 records were excluded, including 449 reviews (including narrative reviews, systematic reviews, and meta-analyses), 5 replies/letters, 108 conference abstracts, 5 case reports, 106 animal studies, 184 registered protocols, 75 non-English publications, 209 duplicate publications, and 2,161 records deemed irrelevant to the research topic. As a result, 76 studies were retained for full-text assessment. After full-text screening, 39 studies were excluded due to ineligible outcome measures (*n* = 23), inappropriate intervention protocols (*n* = 6), non-randomized controlled trial designs (*n* = 6), or unavailable full texts (*n* = 4). Ultimately, 37 studies met the inclusion criteria and were included in the NMA. The study selection process is illustrated in Fig. 1.

### Characteristics of the included studies

A total of 37 RCTs [9–12, 14–46], involving 1,764 patients with stroke and 10 intervention strategies, were included in this NMA. In addition to conventional rehabilitation (C), which was administered as background therapy, the experimental interventions comprised nine NMES-based treatment protocols: NMES alone; gait training (GT) plus NMES; cycling exercise (CE) plus NMES; augmented reality technology (ART) combined with GT and NMES; core stability training (CST) plus NMES; balance training (BT) plus NMES; mirror therapy (MT) plus NMES; motor imagery training (MIT) plus NMES; and proprioceptive neuromuscular facilitation (PNF) plus NMES. All included studies were published after 2000, with intervention durations ranging from 2 to 10 weeks and NMES frequencies ranging from 20 to 80 Hz. The key characteristics of the included studies are summarized in Table 1 and Supplementary Table X. Among the 37 included studies, the availability rates were 26/37 for stimulation frequency, 26/37 for pulse width, 26/37 for stimulation intensity, 8/37 for duty cycle, and 32/37 for trigger mode. The incomplete reporting of these parameters represents a potential source of clinical heterogeneity and may limit the assessment of transitivity across comparisons within the network.

### Risk of bias assessment of the included studies

**Fig. 2:**
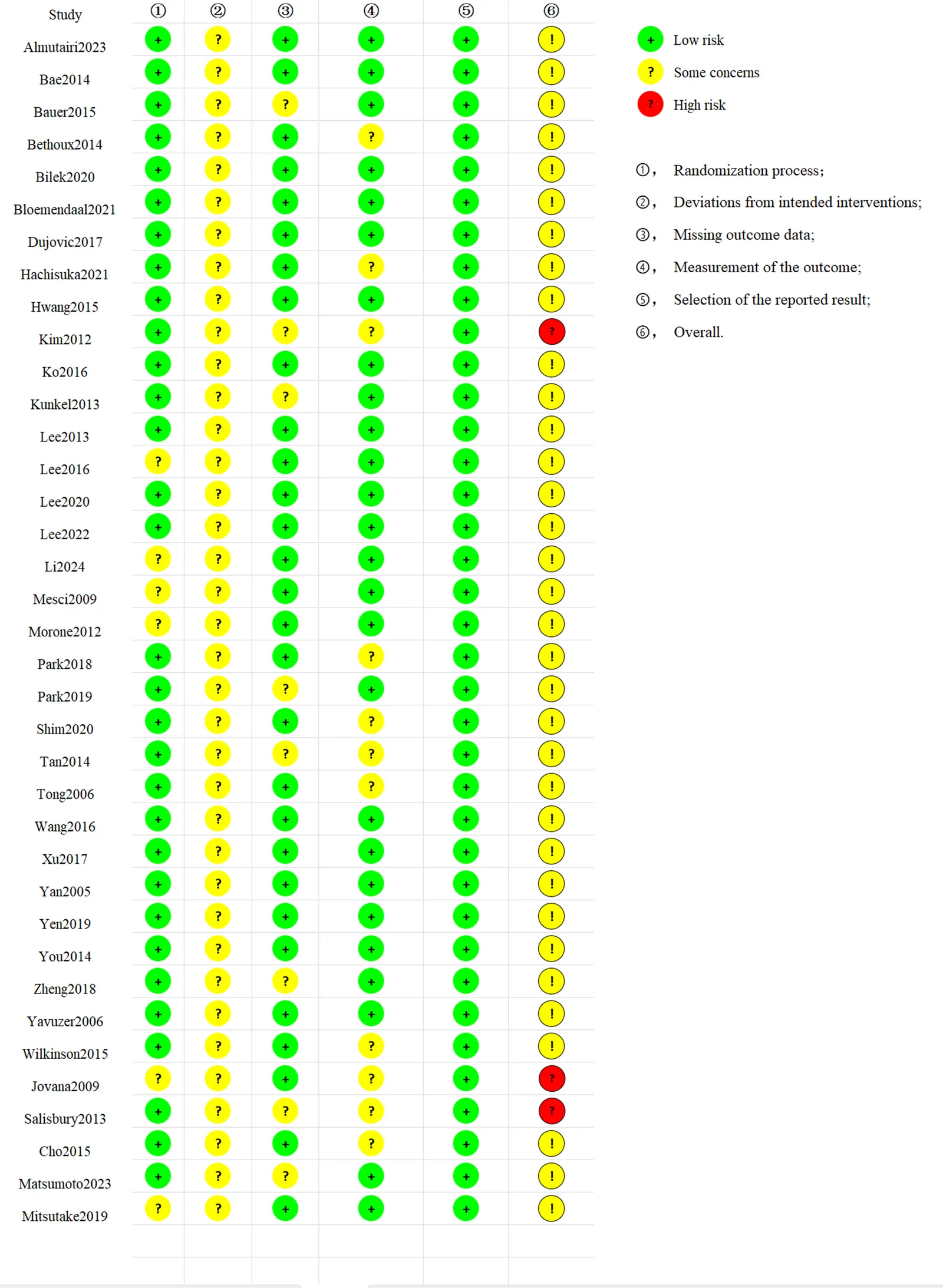
Risk of bias of included studies

Across the 37 RCTs synthesized in this NMA, three were judged to present a high overall risk of bias, while the remaining studies were assessed as raising some concerns. With respect to bias arising from the randomization process, 31 trials were judged to be at low risk, while six were rated as having some concerns, primarily due to inadequate reporting of random sequence generation and insufficient information regarding allocation concealment. Regarding bias related to deviations from intended interventions, all included studies were assessed as having some concerns. This was mainly attributable to the absence of blinding of participants and intervention providers during the treatment period, making it difficult to exclude the potential effects of performance bias on subjective outcome measures or variations in treatment adherence. For bias due to missing outcome data, 29 studies were considered to be at low risk, whereas eight were judged as having some concerns. These concerns largely stemmed from participant attrition leading to incomplete outcome data, with insufficient clarification as to whether the missing data were associated with unfavorable outcomes. In terms of bias in outcome measurement, 26 trials were rated as low risk and 11 as having some concerns. This was mainly because several studies did not clearly report whether outcome assessors were blinded to group allocation, which is particularly relevant given that multiple outcomes in this analysis were assessed using subjective rating scales. All included trials were evaluated as having a low risk of bias in the selection of reported results. Overall, biases related to deviations from intended interventions and outcome measurement were identified as the predominant sources of potential bias in this NMA. A detailed overview of the risk of bias assessments is provided in Fig. 2.

**Table 1:** The basic characteristics of the included studies

NO.	First author	Age (E)	Age (C)	*N* (E)	*N* (C)	Treatment (E)	Treatment (C)	Outcome	Duration (E)	Duration (C)	NMES frequency	Intervention duration	Session frequency
1	Almutairi2023 [9]	57.9 ± 9.9	54.7 ± 12.3	10	9	NMES	C	FAC	32.5 ± 34 m	26.44 ± 30 m	80 Hz	4w	Three times per week
2	Bae2014 [14]	45.4 ± 19.7	52.0 ± 16.1	10	10	GT+NMES	C	BBS, TUGT	9.8 ± 6.0 m	11.5 ± 5.1 m	NR	5w	Three times per week
3	Bauer2015 [15]	59 ± 14	64 ± 11	19	18	CE+NMES	C	FAC	62 ± 43d	42 ± 45d	25 Hz	4w	Three times per week
4	Bethoux2014 [16]	63.87 ± 11.33	64.3 ± 12.01	187	212	GT+NMES	C	BBS, TUGT, 10MWT	6.9 ± 6.43y	6.86 ± 6.64y	NR	6 m	NR
5	Bilek2020 [17]	51.3 ± 3.7	62.6 ± 2.2	30	30	NMES	C	PASS, FAC	NR	NR	50 Hz	6w	Five times per week
6	Bloemendaal2021 [18]	57 ± 8.7	58.7 ± 10.2	19	18	GT+NMES	C	10MWT	16 ± 7.41d	20 ± 5.19d	NR	10w	Five times per week
7	Dujovic2017 [19]	NR	NR	8	8	GT+NMES	C	BBS, FMA-LE,10MWT	NR	NR	40 Hz	4w	Five times per week
8	Hachisuka2021 [20]	61.1 ± 11.8	58.0 ± 13.6	56	58	GT+NMES	C	FMA-LE, 10MWT	69.8 ± 76.9 m	88.4 ± 110.6 m	NR	4w	NR
9	Hwang2015 [21]	50 ± 7.55	49.47 ± 9.01	15	15	GT+NMES	C	BBS, TUGT	192.53 ± 18.79d	194.07 ± 18.95d	25 Hz	4w	Once daily
10	Kim2012 [22]	47.44 ± 8.39	49.11 ± 11.02	9	9	ART+GT+NMES	C	BBS, TUGT	9.74 ± 4.19 m	10.39 ± 3.09 m	35 Hz	8w	Three times per week
		51.5 ± 12.9		10		GT+NMES			9.19 ± 2.74 m		35 Hz	8w	Three times per week
11	Ko2016 [12]	58.5 ± 20	59.5 ± 13.33	10	10	CST+NMES	C	BBS	11 ± 3.33d	12 ± 4.67d	35 Hz	3w	Three times per week
		65.5 ± 14.22		10		NMES			8.5 ± 3.93d		35 Hz	3w	Three times per week
12	Kunkel2013 [23]	64 ± 15	70 ± 10.6	7	7	BT+NMES	C	BBS	4.6w	4.6w	40 Hz	2w	Four times per week
13	Lee2013 [24]	52.47 ± 9.41	56.73 ± 7.24	15	15	GT+NMES	C	BBS, TUGT	4 ± 0.41 m	4.07 ± 1.03 m	NR	4w	Five times per week
14	Lee2016 [25]	55.71 ± 6.7	53.62 ± 6.29	14	13	MT+NMES	C	BBS, TUGT	36.79 ± 26.07 m	42.54 ± 33.9 m	35 Hz	4w	Five times per week
15	Lee2020 [26]	62.16 ± 8.13	64.88 ± 10.35	25	24	BT+NMES	C	BBS, TUGT	16 ± 6.49 m	15.25 ± 6.89 m	30–35 Hz	6w	Five times per week
16	Lee2022 [27]	52.24 ± 11.39	53.24 ± 7.04	17	17	GT+NMES	C	FMA-LE	9.24 ± 2.02 m	9 ± 1.54 m	33 Hz	4w	Five times per week
17	Li2024 [28]	41.77 ± 3.56	42.68 ± 3.97	60	60	CST+NMES	C	BBS, TUGT, FAC	15.33 ± 1.46 m	15.57 ± 1.61 m	80 Hz	8w	Five times per week
18	Mesci2009 [29]	62.65 ± 7.52	59.1 ± 8.58	20	20	NMES	C	BS-LE, FAC	9.45 ± 4.8 m	7.3 ± 4.42 m	50 Hz	4w	Five times per week
19	Morone2012 [30]	61.2 ± 16.2	53.3 ± 14.6	10	10	GT+NMES	C	FAC	27 ± 27d	13 ± 7d	NR	4w	Five times per week
20	Park2018 [31]	68.6 ± 13.57	57.5 ± 11.84	10	10	CST+NMES	C	BBS	17.3 ± 9.12d	13.6 ± 5.52d	35 Hz	3w	Five times per week
21	Park2019 [11]	70.13 ± 7.56	65.86 ± 13.10	10	10	MIT+NMES	C	FMA-LE	3.25 ± 1.66 m	3.43 ± 0.97 m	NR	4w	Five times per week
22	Shim2020 [32]	59.65 ± 16.52	56 ± 15.61	17	16	PNF+NMES	C	BBS	11.59 ± 5.9 m	13.88 ± 5.51 m	35 Hz	4w	Five times per week
23	Tan2014 [10]	63.4 ± 10.6	67 ± 9	16	15	NMES	C	BBS, FAC	41.3 ± 29.4d	41.5 ± 20.4d	30 Hz	3w	Five times per week
24	Tong2006 [33]	61.8 ± 10.8	66.1 ± 9.9	15	15	GT+NMES	C	BBS, FMA-LE, FAC	2.3 ± 1w	2.7 ± 1.3w	NR	4w	Five times per week
25	Wang2016 [34]	50.17 ± 9.8	51.81 ± 10.41	17	16	NMES	C	TUGT	28.71 ± 8.12d	29.88 ± 9.42d	20 Hz	4w	Ten times per week
26	Xu2017 [35]	55 ± 10.98	56.09 ± 8.12	23	23	MT+NMES	C	BS-LE	43.25 ± 5.95d	45.78 ± 6.5d	50 Hz	4w	Five times per week
27	Yan2005 [36]	68.2 ± 7.7	70.4 ± 7.6	13	13	NMES	C	TUGT	8.7 ± 5.8d	9.1 ± 3.5d	30 Hz	3w	Five times per week
28	Yen2019 [37]	61.62 ± 9.31	61.42 ± 12.61	13	14	NMES	C	PASS	1.38 ± 0.51d	1.36 ± 0.5d	30 Hz	2w	Five times per week
29	You2014 [38]	60.8 ± 10.8	64.1 ± 9.7	19	18	NMES	C	BBS, FMA-LE	25.9 ± 21.3d	22.7 ± 16.6d	30 Hz	3w	Five times per week
30	Zheng2018 [39]	59 ± 11	59 ± 9	18	15	NMES	C	BBS, FMA-LE	20 ± 11d	20 ± 12d	30 Hz	3w	Five times per week
31	Yavuzer2006 [40]	56.3 ± 7.5	54.2 ± 8.1	12	13	NMES	C	BS-LE	2.4 ± 1.7 m	2.3 ± 1.3 m	80 Hz	4w	Five times per week
32	Wilkinson2015 [41]	65.4 ± 8.75	64.5 ± 10.25	10	10	NMES	C	10MWT	10.8 ± 3.15w	9.9 ± 4.13w	NR	6w	Twice per week
33	Jovana2009 [42]	61 ± 13.1	57.2 ± 13.3	7	6	GT+NMES	C	FMA-LE	22.6 ± 7.6d	22.7 ± 5.3d	50 Hz	4w	Five times per week
34	Salisbury2013 [43]	55.8 ± 11.3	52,6 ± 17.2	9	7	GT+NMES	C	FAC	51.7 ± 34.6d	69 ± 35.5d	NR	6w	Five times per week
35	Cho2015 [44]	57 ± 9.1	57.8 ± 7.9	10	11	GT+NMES	C	BBS	22.5 ± 12.6 m	21.6 ± 6.7 m	40 Hz	4w	Five times per week
36	Matsumoto2023 [45]	63.5 ± 10.5	64.3 ± 11.8	92	92	NMES	C	FMA-LE, 10MWT	59.5 ± 32.6d	63.7 ± 30.4d	NR	8w	Five times per week
37	Mitsutake2019 [46]	68.75 ± 10.59	74.69 ± 15.13	12	13	NMES	C	10MWT	43.17 ± 35.26d	49.69 ± 31.16d	20 Hz	2w	Seven times per week

E: experimental group; C: control group; y: year; m: month; w: week; d: day; NMES: neuromuscular electrical stimulation; C: conventional rehabilitation; GT: gait training; CE: cycling exercise; ART: augmented reality technology; CST: core stability training; BT: balance training; MT: mirror therapy; MIT: motor imagery training; PNF: proprioceptive neuromuscular facilitation; BBS: Berg Balance Scale; PASS: Postural Assessment Scale for Stroke Patients; BS-LE: Brunnstrom Stages–Lower Extremity; FAC: Functional Ambulation Category Scale; 10MWT: 10-Meter Walking Test; TUGT: Timed Up and Go Test; FMA-LE: Fugl-Meyer Assessment–Lower Extremity; NR: not reported; Hz: Hertz

### Network meta-analysis

**Fig. 3:**
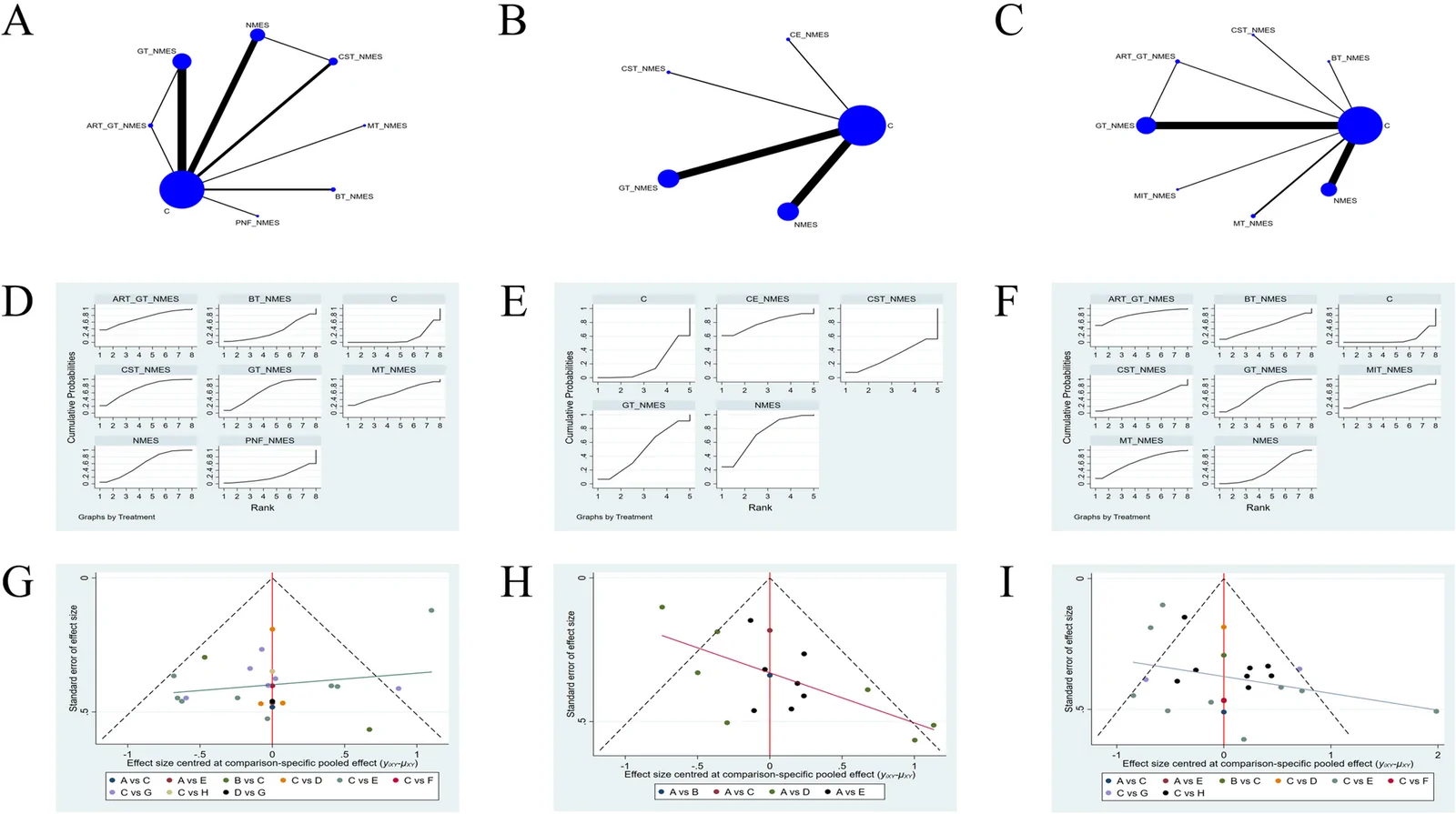
Network meta-analysis graph group. **A** Network plot for balance ability. **B** Network plot for walking ability. **C** Network plot for lower limb functional mobility. **D** Cumulative ranking probability plot for balance ability. **E** Cumulative ranking probability plot for walking ability. **F** Cumulative ranking probability plot for lower limb functional mobility. **G** Funnel plot for balance ability. **H** Funnel plot for walking ability. **I** Funnel plot for lower limb functional mobility. NMES: neuromuscular electrical stimulation; C: conventional rehabilitation; GT: gait training; CE: cycling exercise; ART: augmented reality technology; CST: core stability training; BT: balance training; MT: mirror therapy; MIT: motor imagery training; PNF: proprioceptive neuromuscular facilitation. Node sizes are proportional to the total number of participants, and edge widths are proportional to the number of studies informing each direct comparison. The number of studies and participants for each node are detailed in Supplementary Table Y

Figure 3A–C illustrate the evidence networks constructed for balance ability, walking ability, and lower limb functional mobility. These visualizations summarize the pattern of direct evidence available among the evaluated intervention strategies. Across all three outcomes, the networks were predominantly star-shaped, with the majority of direct comparisons involving C as one arm. Only two head-to-head comparisons between active NMES-based interventions were available (ART+GT+NMES vs. GT+NMES and CST+NMES vs. NMES), both limited to specific outcomes. As a result, most relative effect estimates between active interventions were derived primarily from indirect evidence through the common comparator. Individual interventions are depicted as nodes, with their relative sizes reflecting the total number of participants contributing data to each strategy. Connections between nodes represent head-to-head comparisons, and the width of each connection indicates the strength of direct evidence, as determined by the number of studies informing that comparison.

#### Balance ability

The network plot for the balance ability outcome is presented in Fig. 3A. A total of 20 studies [10, 12, 14, 16, 17, 19, 21–26, 28, 31–33, 37–39, 44] reported balance ability in stroke patients, involving eight intervention strategies: ART+GT+NMES, GT+NMES, NMES, CST+NMES, MT+NMES, BT+NMES, PNF+NMES, and C. All interventions were directly compared with C. In addition, ART+GT+NMES was directly compared with GT+NMES, and NMES was directly compared with CST+NMES, resulting in two closed loops in the network plot. Under the consistency model, a random-effects NMA was performed to synthesize direct and indirect evidence, with an estimated τ of 0.53 (95% CrI: 0.31–0.84).

The league table for the balance ability outcome is shown in the lower-left part of Table 2. According to the table, CST+NMES (SMD = 0.99, 95% CrI: 0.33–1.67), GT+NMES (SMD = 0.83, 95% CrI: 0.35–1.27), and NMES (SMD = 0.71, 95% CrI: 0.21–1.22) were more effective than C, with all 95% CrIs excluding zero.

The SUCRA ranking table for the balance ability outcome is presented in Table 4, and the corresponding cumulative ranking probability plot is shown in Fig. 3D. As indicated by Table 4; Fig. 3D, the interventions rank as follows: CST+NMES (SUCRA = 76.02%) > ART+GT+NMES (SUCRA = 74.35%) > GT+NMES (SUCRA = 65.22%) > MT+NMES (SUCRA = 59.37%) > NMES (SUCRA = 56.55%) > BT+NMES (SUCRA = 32.62%) > PNF+NMES (SUCRA = 23.39%) > C (SUCRA = 12.49%).

In summary, CST+NMES, GT+NMES, and NMES showed statistically significant improvements compared with C. In terms of ranking probability, CST+NMES (SUCRA = 76.02%) ranked highest, followed by ART+GT+NMES (SUCRA = 74.35%) and GT+NMES (SUCRA = 65.22%). However, as the credible intervals of these interventions overlapped considerably, the ranking differences should be interpreted with caution. In addition, it should be noted that several interventions in this network, including ART+GT+NMES, MT+NMES, and PNF+NMES, were each informed by only a single study, which limits the reliability of their ranking positions (Supplementary Table Y).

#### Walking ability

The network plot for the walking ability outcome is presented in Fig. 3B. A total of 16 studies [9, 10, 15–20, 28–30, 33, 41, 43, 45, 46] reported walking ability in stroke patients, involving five intervention strategies, including CST+NMES, CE+NMES, NMES, GT+NMES, and C. For the walking ability outcome, all interventions were directly compared with C. Under the consistency model, a random-effects NMA was performed to synthesize direct and indirect evidence, with an estimated τ of 0.46 (95% CrI: 0.20–0.86).

The league table for walking ability is shown in the upper right section of Table 2. As indicated in this section, no meaningful differences were identified between any intervention comparisons within the network, as all 95% CrIs included zero.

The SUCRA ranking table for walking ability is presented in Table 4, and the corresponding cumulative ranking probability plot is shown in Fig. 3E. According to Table 4; Fig. 3E, the ranking of interventions was as follows: CE+NMES (SUCRA = 77.46%) > NMES (SUCRA = 70.93%) > GT+NMES (SUCRA = 49.29%) > CST+NMES (SUCRA = 32.44%) > C (SUCRA = 19.89%).

In summary, no statistically significant differences were observed among the NMES-based interventions for walking ability, and the credible intervals for all comparisons overlapped substantially. The SUCRA values provide supplementary information on ranking probabilities but should not be interpreted as evidence of differential efficacy in the absence of significant effect estimates [47]. Therefore, while CE+NMES received the highest SUCRA value (77.46%), this ranking should be regarded as exploratory and interpreted with considerable caution. Notably, CE+NMES was represented by only a single trial in this network, further limiting the certainty of its ranking position (Supplementary Table Y). Further trials with larger sample sizes are needed to determine whether CE+NMES offers meaningful benefits for walking recovery after stroke.

**Table 2:** League table of balance ability and walking ability

	Walking ability								
**Balance Ability**	**ART+GT+NMES**								
	0.72 (− 0.68, 2.13)	**BT+NMES**							
	1.02 (− 0.02, 2.04)	0.3 (− 0.68, 1.24)	**C**	0.66 (− 0.54, 1.85)	0.03 (− 1.04, 1.09)	0.22 (− 0.21, 0.75)		0.42 (− 0.02, 0.88)	
				**CE+NMES**	− 0.63 (− 2.22, 0.97)	− 0.45 (− 1.66, 0.91)		− 0.24 (− 1.5, 1.05)	
	0.03 (− 1.21, 1.24)	− 0.69 (− 1.89, 0.46)	− 0.99 (− 1.67, − 0.33)		**CST+NMES**	0.18 (− 0.9, 1.43)		0.4 (− 0.75, 1.56)	
	0.2 (− 0.83, 1.24)	− 0.52 (− 1.58, 0.53)	− 0.83 (− 1.27, − 0.35)		0.16 (− 0.62, 1.01)	**GT+NMES**		0.21 (− 0.51, 0.81)	
	0.25 (− 1.45, 1.92)	− 0.47 (− 2.13, 1.14)	− 0.78 (− 2.11, 0.56)		0.22 (− 1.27, 1.71)	0.05 (− 1.38, 1.44)	**MT+NMES**		
	0.31 (− 0.85, 1.44)	− 0.41 (− 1.51, 0.66)	− 0.71 (− 1.22, − 0.21)		0.28 (− 0.48, 1.05)	0.12 (− 0.59, 0.78)	0.06 (− 1.36, 1.49)	**NMES**	
	0.96 (− 0.69, 2.59)	0.24 (− 1.37, 1.81)	− 0.06 (− 1.33, 1.21)		0.93 (− 0.51, 2.37)	0.77 (− 0.61, 2.1)	0.71 (− 1.14, 2.55)	0.65 (− 0.71, 2.01)	**PNF+NMES**

NMES: neuromuscular electrical stimulation; C: conventional rehabilitation; GT: gait training; CE: cycling exercise; ART: augmented reality technology; CST: core stability training; BT: balance training; MT: mirror therapy; PNF: proprioceptive neuromuscular facilitation

**Table 3:** League table of lower limb functional mobility

Lower limb functional mobility							
**ART+GT+NMES**	− 0.77 (− 2.56, 1.01)	− 1.38 (− 2.52, − 0.25)	− 0.9 (− 2.65, 0.82)	− 0.53 (− 1.65, 0.62)	− 0.68 (− 2.61, 1.23)	− 0.44 (− 1.97, 1.08)	− 0.83 (− 2.07, 0.4)
	**BT+NMES**	− 0.61 (− 1.99, 0.77)	− 0.14 (− 2.03, 1.77)	0.24 (− 1.2, 1.73)	0.08 (− 1.98, 2.15)	0.33 (− 1.39, 2.03)	− 0.07 (− 1.53, 1.41)
		**C**	0.48 (− 0.83, 1.79)	0.85 (0.39, 1.36)	0.69 (− 0.84, 2.24)	0.94 (− 0.08, 1.95)	0.55 (0.06, 1.05)
			**CST+NMES**	0.37 (− 0.99, 1.8)	0.21 (− 1.8, 2.24)	0.46 (− 1.2, 2.12)	0.07 (− 1.32, 1.48)
				**GT+NMES**	− 0.16 (− 1.79, 1.44)	0.09 (− 1.06, 1.18)	− 0.3 (− 1.02, 0.38)
					**MIT+NMES**	0.25 (− 1.6, 2.09)	− 0.14 (− 1.76, 1.48)
						**MT+NMES**	− 0.39 (− 1.51, 0.75)
							**NMES**

NMES: neuromuscular electrical stimulation; C: conventional rehabilitation; GT: gait training; ART: augmented reality technology; CST: core stability training; BT: balance training; MT: mirror therapy; MIT: motor imagery training

**Table 4:** SUCRA ranking table

Treatment	Balance Ability	Walking Ability	Lower Limb Functional Mobility
ART+GT+NMES	74.35	NR	84.23
BT+NMES	32.62	NR	46.93
C	12.49	19.89	8.98
CST+NMES	76.02	32.44	39.82
GT+NMES	65.22	49.29	62.22
MIT+NMES	NR	NR	50.79
MT+NMES	59.37	NR	65.24
NMES	56.55	70.93	41.78
CE+NMES	NR	77.46	NR
PNF+NMES	23.39	NR	NR

NMES: neuromuscular electrical stimulation; C: conventional rehabilitation; GT: gait training; CE: cycling exercise; ART: augmented reality technology; CST: core stability training; BT: balance training; MT: mirror therapy; MIT: motor imagery training; PNF: proprioceptive neuromuscular facilitation; NR: not reported

#### Lower limb functional mobility

The network plot for lower limb functional mobility is presented in Fig. 3C. A total of 22 studies [11, 14, 16, 19–22, 24–29, 33–36, 38–40, 42, 45] reported outcomes related to lower limb functional mobility in patients with stroke, involving eight intervention strategies: ART+GT+NMES, CST+NMES, BT+NMES, NMES, MT+NMES, MIT+NMES, GT+NMES, and C. For the lower limb functional mobility outcome, all interventions were directly compared with C. In addition, ART+GT+NMES was directly compared with GT+NMES, resulting in one closed loop in the network plot. Under the consistency model, a random-effects NMA was performed to synthesize direct and indirect evidence, with an estimated τ of 1.05 (95% CrI: 0.69–1.57).

The league table for lower limb functional mobility is presented in Table 3. As shown in Table 3, ART+GT+NMES (SMD = 1.38, 95% CrI: 0.25–2.52), GT+NMES (SMD = 0.85, 95% CrI: 0.39–1.36), and NMES (SMD = 0.55, 95% CrI: 0.06–1.05) demonstrated greater improvements in lower limb functional mobility compared with C, with all 95% CrIs excluding zero.

The SUCRA ranking table for lower limb functional mobility is shown in Table 4, and the corresponding cumulative ranking probability plot is presented in Fig. 3F. As illustrated in Table 4; Fig. 3F, the interventions were ranked as follows: ART+GT+NMES (SUCRA = 84.23%) > MT+NMES (SUCRA = 65.24%) > GT+NMES (SUCRA = 62.22%) > MIT+NMES (SUCRA = 50.79%) > BT+NMES (SUCRA = 46.93%) > NMES (SUCRA = 41.78%) > CST+NMES (SUCRA = 39.82%) > C (SUCRA = 8.98%).

In summary, ART+GT+NMES, GT+NMES, and NMES demonstrated statistically significant improvements in lower limb functional mobility compared with C. In terms of ranking probability, ART+GT+NMES (SUCRA = 84.23%) ranked highest, followed by MT+NMES (SUCRA = 65.24%) and GT+NMES (SUCRA = 62.22%). Notably, while MT+NMES ranked second by SUCRA, its comparison with C did not reach statistical significance, and this ranking should therefore be interpreted cautiously. Furthermore, ART+GT+NMES, BT+NMES, and MIT+NMES were each supported by only one study, and their high SUCRA rankings may not be robust to the addition of further evidence (Supplementary Table Y).

#### Two-dimensional cluster analysis

**Fig. 4:**
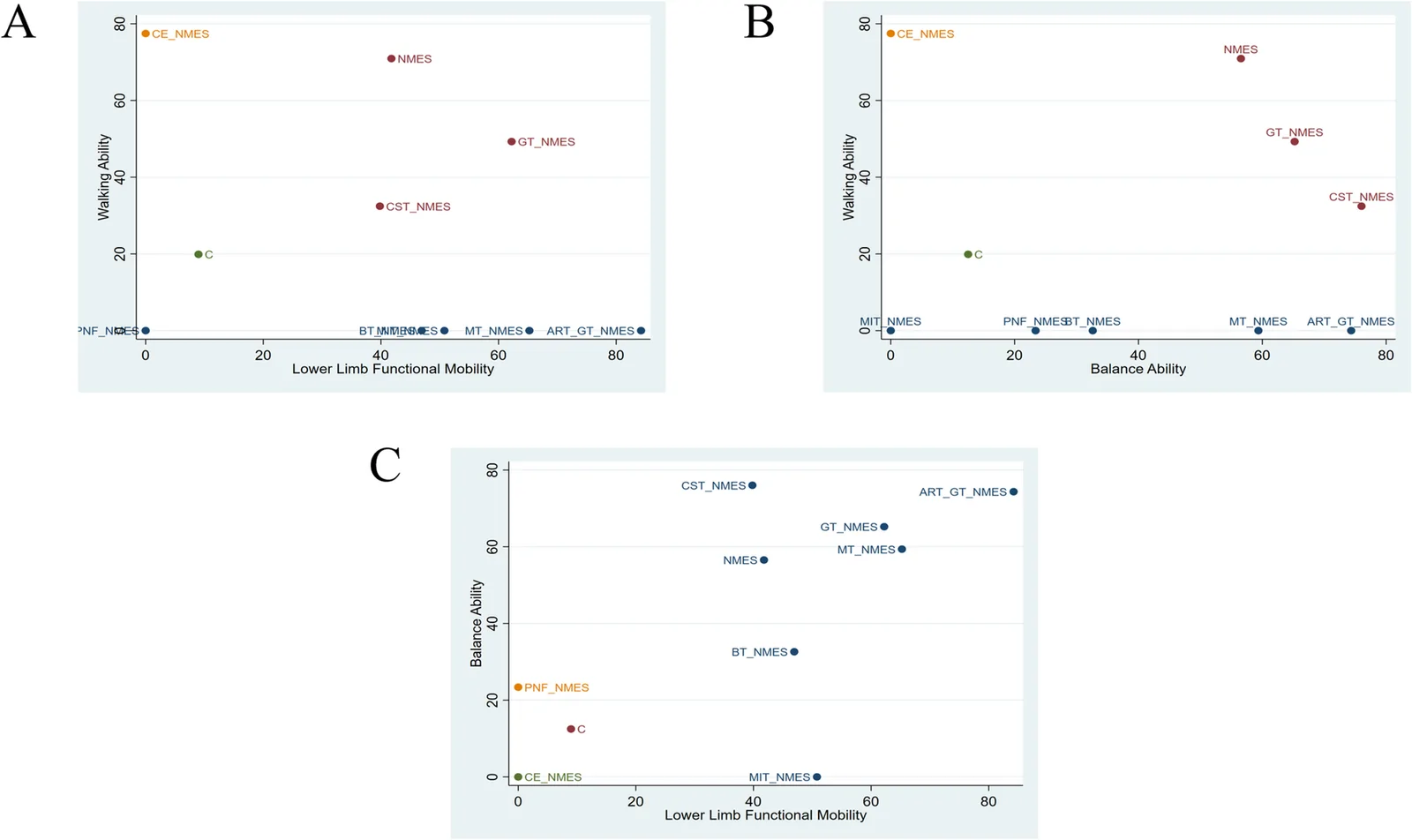
Two-dimensional cluster ranking plot. **A** Two-dimensional cluster ranking plot for walking ability and lower limb functional mobility. **B** Two-dimensional cluster ranking plot for walking ability and balance ability. **C** Two-dimensional cluster ranking plot for balance ability and lower limb functional mobility. NMES: neuromuscular electrical stimulation; C: conventional rehabilitation; GT: gait training; CE: cycling exercise; ART: augmented reality technology; CST: core stability training; BT: balance training; MT: mirror therapy; MIT: motor imagery training; PNF: proprioceptive neuromuscular facilitation

The two-dimensional cluster ranking plots integrating SUCRA values across outcomes are presented in Figs. 4A–C, illustrating the comparative performance of NMES-based combination strategies across multiple functional domains. When walking ability and lower limb functional mobility were jointly considered (Fig. 4A), GT+NMES (SUCRA = 49.29%/62.22%) occupied a favorable position, indicating balanced effectiveness across both outcomes. For the joint evaluation of walking ability and balance ability (Fig. 4B), both GT+NMES (SUCRA = 49.29%/65.22%) and NMES (SUCRA = 70.93%/56.55%) demonstrated comparatively strong performance, suggesting their potential advantages in addressing these functional domains. In contrast, when balance ability and lower limb functional mobility were analyzed together (Fig. 4C), ART+GT+NMES (SUCRA = 84.23%/74.35%), GT+NMES (SUCRA = 65.22%/62.22%), and MT+NMES (SUCRA = 59.37%/65.24%) clustered toward the region associated with higher SUCRA values, reflecting more consistent effects across the two outcomes. Across the three functional dimensions examined—balance ability, walking ability, and lower limb functional mobility—GT+NMES (SUCRA = 65.22%/49.29%/62.22%) consistently demonstrated a stable and favorable ranking profile. This pattern indicates that GT+NMES may offer a comparatively robust intervention option for improving lower limb function after stroke when multiple rehabilitation outcomes are considered simultaneously.

### Subgroup analysis

Subgroup analyses were conducted according to the time since stroke onset. Patients were categorized into three subgroups: acute phase (time since onset < 1 month), subacute phase (time since onset between 1 and 6 months), and chronic phase (time since onset > 6 months).

#### Acute phase

##### Balance ability

The network plot for balance ability in acute stroke patients is shown in Supplementary Fig. 1. Six studies reported balance ability in acute stroke patients, involving four interventions: GT+NMES, CST+NMES, NMES, and C.

The league table for balance ability in acute stroke patients is presented in Supplementary Table 2. According to Supplementary Table 2, CST+NMES (SMD = 1.07, 95% CrI: 0.19–1.95) and NMES (SMD = 0.72, 95% CrI: 0.05–1.36) were superior to C, with both 95% CrIs excluding zero.

The SUCRA ranking table for balance ability in acute stroke patients is presented in Supplementary Table 3. According to Supplementary Table 3, CST+NMES (SUCRA = 89.78%) > NMES (SUCRA = 66.92%) > GT+NMES (SUCRA = 28.3%) > C (SUCRA = 15.00%).

##### Walking ability

Only three studies reported walking ability in acute stroke patients. In these studies, the intervention group was GT+NMES and the control group was C. Since only two interventions were involved, a network could not be formed, and network meta-analysis was not applicable. Therefore, a pairwise meta-analysis was conducted to evaluate the efficacy of GT+NMES compared with C.

The forest plot for walking ability in acute stroke patients is shown in Supplementary Fig. 2. As shown in Supplementary Fig. 2, GT+NMES (SMD = 0.68, 95% CI: -0.31–1.67) was superior to C; however, the 95% CI included zero, indicating that the difference was not statistically significant.

##### Lower limb functional mobility

The network plot for lower limb functional mobility in acute stroke patients is shown in Supplementary Fig. 3. Five studies reported lower limb functional mobility in acute stroke patients, involving three interventions: GT+NMES, NMES, and C.

The league table for lower limb functional mobility in acute stroke patients is shown in Supplementary Table 4. As shown in Supplementary Table 4, no meaningful differences in treatment effects were observed among the interventions, as all 95% CrIs included zero.

The SUCRA ranking for lower limb functional mobility in acute stroke patients is shown in Supplementary Table 3. GT+NMES (SUCRA = 84.43%) ranked highest, followed by NMES (SUCRA = 59.71%) and C (SUCRA = 5.86%).

#### Subacute phase

##### Balance ability

The network plot for balance ability in subacute stroke patients is shown in Supplementary Fig. 4. Three studies reported balance ability in subacute stroke patients, involving four interventions: BT+NMES, GT+NMES, NMES, and C.

The league table for balance ability in subacute stroke patients is shown in Supplementary Table 5. As shown in Supplementary Table 5, no meaningful differences in treatment effects were observed among the interventions, as all 95% CrIs included zero.

The SUCRA ranking for balance ability in subacute stroke patients is shown in Supplementary Table 6. NMES (SUCRA = 87.04%) ranked highest, followed by GT+NMES (SUCRA = 60.83%), C (SUCRA = 31.57%), and BT+NMES (SUCRA = 20.56%).

##### Walking ability

The network plot for walking ability in subacute stroke patients is shown in Supplementary Fig. 5. Six studies reported walking ability in subacute stroke patients, involving four interventions: CE+NMES, GT+NMES, NMES, and C.

The league table for walking ability in subacute stroke patients is shown in Supplementary Table 7. As shown in Supplementary Table 7, no meaningful differences in treatment effects were observed among the interventions, as all 95% CrIs included zero.

The SUCRA ranking for walking ability in subacute stroke patients is shown in Supplementary Table 6. CE+NMES (SUCRA = 82.72%) ranked highest, followed by NMES (SUCRA = 66.08%), GT+NMES (SUCRA = 31.54%), and C (SUCRA = 19.67%).

##### Lower limb functional mobility

The network plot for lower limb functional mobility in subacute stroke patients is shown in Supplementary Fig. 6. Six studies reported lower limb functional mobility in subacute stroke patients, involving five interventions: GT+NMES, MIT+NMES, MT+NMES, NMES, and C.

The league table for lower limb functional mobility in subacute stroke patients is shown in Supplementary Table 8. As shown in Supplementary Table 8, MT+NMES (SMD = 1.63, 95% CrI: 0–3.26) was superior to C, with the 95% CrI marginally excluding zero.

The SUCRA ranking for lower limb functional mobility in subacute stroke patients is shown in Supplementary Table 6. MT+NMES (SUCRA = 81.27%) ranked highest, followed by GT+NMES (SUCRA = 79.64%), MIT+NMES (SUCRA = 45.37%), NMES (SUCRA = 35.58%), and C (SUCRA = 8.14%).

#### Chronic phase

##### Balance ability

The network plot for balance ability in chronic stroke patients is shown in Supplementary Fig. 7. Nine studies reported balance ability in chronic stroke patients, involving seven interventions: ART+GT+NMES, CST+NMES, BT+NMES, PNF+NMES, MT+NMES, GT+NMES, and C.

The league table for balance ability in chronic stroke patients is shown in Supplementary Table 9. As shown in Supplementary Table 9, no meaningful differences were observed between interventions, as all 95% CrIs included zero.

The SUCRA ranking for balance ability in chronic stroke patients is shown in Supplementary Table 10. MT+NMES (SUCRA = 88.91%) ranked highest, followed by CST+NMES (SUCRA = 72.34%), ART+GT+NMES (SUCRA = 52.58%), GT+NMES (SUCRA = 49.57%), BT+NMES (SUCRA = 35.47%), PNF+NMES (SUCRA = 34.74%), and C (SUCRA = 16.38%).

##### Walking ability

The network plot for walking ability in chronic stroke patients is shown in Supplementary Fig. 8. Five studies reported walking ability in chronic stroke patients, involving four interventions: GT+NMES, CST+NMES, NMES, and C.

The league table for walking ability in chronic stroke patients is shown in Supplementary Table 11. As shown in Supplementary Table 11, no meaningful differences were observed between interventions, as all 95% CrIs included zero.

The SUCRA ranking for walking ability in chronic stroke patients is shown in Supplementary Table 10. NMES (SUCRA = 84.07%) ranked highest, followed by CST+NMES (SUCRA = 54.21%), C (SUCRA = 51.91%), and GT+NMES (SUCRA = 9.81%).

##### Lower limb functional mobility

The network plot for lower limb functional mobility in chronic stroke patients is shown in Supplementary Fig. 9. Ten studies reported lower limb functional mobility in chronic stroke patients, involving seven interventions: ART+GT+NMES, CST+NMES, BT+NMES, GT+NMES, MT+NMES, NMES, and C.

The league table for lower limb functional mobility in chronic stroke patients is shown in Supplementary Table 12. As shown in Supplementary Table 12, no meaningful differences were observed between interventions, as all 95% CrIs included zero.

The SUCRA ranking for lower limb functional mobility in chronic stroke patients is shown in Supplementary Table 10. ART+GT+NMES (SUCRA = 77.00%) ranked highest, followed by GT+NMES (SUCRA = 60.57%), NMES (SUCRA = 59.88%), BT+NMES (SUCRA = 49.84%), CST+NMES (SUCRA = 45.19%), MT+NMES (SUCRA = 36.11%), and C (SUCRA = 21.40%).

In summary, stroke patients at different stages of onset respond differently to various NMES-based combination interventions. Although some comparisons between interventions did not reach statistical significance, SUCRA, which is based on overall ranking probabilities, can still reflect the relative advantages of each intervention in multiple comparisons [47]. For lower limb function in acute stroke patients, CST+NMES may demonstrate better efficacy; for subacute stroke patients, GT+NMES may show better effects; and for chronic stroke patients, ART+GT+NMES may exhibit better efficacy.

### Global consistency and local inconsistency assessment

Consistency within the evidence networks was examined using both global and local approaches across all three outcomes. At the global level, comparison of DIC values between consistency and inconsistency models revealed minimal differences for balance ability (39.93 vs. 41.40), walking ability (both 28.37), and lower limb functional mobility (44.23 vs. 44.95), with all differences remaining below the predefined threshold of 5. These findings indicate satisfactory agreement between direct and indirect evidence at the network level for each outcome. For outcomes in which closed loops were present, additional node-splitting analyses were undertaken to explore potential local inconsistency. Balance ability contained two closed loops, and the corresponding node-splitting results (Supplementary Figs. 10 and 11) demonstrated no statistically significant discrepancies between direct and indirect comparisons across all contrasts (*p* > 0.05). Similarly, lower limb functional mobility included one closed loop, and node-splitting analysis (Supplementary Fig. 12) showed no significant inconsistency between sources of evidence (*p* > 0.05). In contrast, the network for walking ability did not contain any closed loops, and therefore local inconsistency could not be evaluated for this outcome. The absence of closed loops means that all effect estimates for walking ability were derived entirely from indirect comparisons through the common comparator, and the agreement between direct and indirect evidence could not be verified. Consequently, the treatment rankings for walking ability should be interpreted as exploratory. Taken together, the consistency assessments at both global and local levels provide no indication of meaningful inconsistency within the networks, supporting the robustness of the pooled effect estimates and the reliability of intervention rankings derived from this NMA.

### Sensitivity analysis

A sensitivity analysis was conducted by excluding studies rated as high overall risk of bias under the ROB 2.0 framework (Kim 2012, Jovana 2009, and Salisbury 2013) from the relevant outcome networks.

For balance ability, Kim 2012 was excluded, resulting in the loss of the ART+GT+NMES node (as this was the only study informing that node). The reanalyzed SUCRA ranking order was: CST+NMES (SUCRA = 78.68%) > GT+NMES (SUCRA = 76.54%) > MT+NMES (SUCRA = 61.53%) > NMES (SUCRA = 59.18%) > BT+NMES (SUCRA = 34.99%) > PNF+NMES (SUCRA = 24.94%) > C (SUCRA = 12.49%). The ranking order remained consistent with the main analysis, indicating good robustness of the balance ability findings.

For walking ability, Salisbury 2013 was excluded. The reanalyzed SUCRA ranking order was: CE+NMES (SUCRA = 76.1%) > NMES (SUCRA = 69.43%) > GT+NMES (SUCRA = 52.14%) > CST+NMES (SUCRA = 32.58%) > C (SUCRA = 19.75%). The ranking order was consistent with the main analysis, confirming the robustness of the walking ability results.

For lower limb functional mobility, Kim 2012 and Jovana 2009 were excluded, resulting in the loss of the ART+GT+NMES node. The reanalyzed SUCRA ranking order was: GT+NMES (SUCRA = 80.52%) > MT+NMES (SUCRA = 59.7%) > MIT+NMES (SUCRA = 51.14%) > BT+NMES (SUCRA = 48.41%) > NMES (SUCRA = 47.1%) > CST+NMES (SUCRA = 44.18%) > C (SUCRA = 18.95%). After excluding the high-risk studies, the SUCRA value of GT+NMES increased substantially (from 62.22% to 80.52%), suggesting that its therapeutic efficacy in the main analysis may have been underestimated. The remaining interventions showed generally stable SUCRA values and ranking positions, indicating overall robustness of the findings for this outcome.

### Confidence in network meta-analysis (CINeMA)

The CINeMA assessment results for all pairwise comparisons across the three outcomes are presented in Supplementary Table 13 (balance ability), Supplementary Table 14 (walking ability), and Supplementary Table 15 (lower limb functional mobility).

For balance ability, 26 of 28 comparisons (92.9%) were rated as moderate confidence and 2 (7.1%) as low confidence. The predominant reasons for downgrading were heterogeneity (for comparisons informed by multiple studies) and imprecision (for comparisons informed by limited or indirect evidence only). The two low-confidence comparisons were downgraded due to concurrent concerns regarding within-study bias and imprecision.

For walking ability, 9 of 10 comparisons (90%) were rated as low confidence and 1 (10%) as moderate. All comparisons were downgraded for incoherence, reflecting the absence of closed loops in this network and the consequent inability to verify agreement between direct and indirect evidence. This finding is consistent with the exploratory characterization of walking ability rankings described in Sect.  3.6.

For lower limb functional mobility, 1 of 28 comparisons (3.6%) was rated as high confidence, 26 (92.9%) as moderate, and 1 (3.6%) as low. The predominant reasons for downgrading were heterogeneity and imprecision. Overall, the evidence confidence for lower limb functional mobility was the most favorable among the three outcomes.

### Publication bias

Publication bias was examined for balance ability, walking ability, and lower limb functional mobility, with the corresponding funnel plots presented in Figs. 3G–I. Egger’s and Begg’s tests were further conducted to quantitatively assess publication bias across outcomes. For balance ability, Begg’s test showed no significant publication bias (*p* = 0.882), whereas Egger’s test indicated significant publication bias (*p* < 0.001). For walking ability, Begg’s test did not detect significant publication bias (*p* = 0.105), while Egger’s test suggested significant publication bias (*p* < 0.001). Similarly, for lower-limb functional mobility, Begg’s test showed no significant publication bias (*p* = 0.982), whereas Egger’s test indicated significant publication bias (*p* < 0.001). Overall, although Begg’s test did not reveal significant publication bias, Egger’s regression test suggested potential small-study effects or publication bias across all three outcomes.

## Discussion

The evidence base synthesized in this NMA comprised 37 randomized controlled trials, covering 1,764 individuals with stroke and encompassing nine distinct NMES-based combination strategies. The results indicated that different NMES combination therapies had varying efficacy in improving lower limb dysfunction in stroke patients. CST+NMES received the highest ranking probability for improving lower limb balance, CE+NMES ranked highest for walking ability (though without statistically significant effect estimates, and with all rankings for this outcome derived entirely from indirect evidence in a network without closed loops), and ART+GT+NMES ranked highest for improving lower limb functional mobility. A comprehensive assessment revealed that GT+NMES demonstrated relatively stable ranking performance across all three lower limb function outcomes, suggesting that it warrants further investigation as a promising NMES-based combination strategy for stroke patients with lower limb dysfunction. Additionally, this NMA found that the efficacy of different NMES combination interventions varied depending on the stroke phase. CST+NMES appeared to be more effective for patients in the acute phase, GT+NMES for those in the subacute phase, and ART+GT+NMES for patients in the chronic phase.

Multiple previous systematic reviews and meta-analyses have consistently demonstrated that NMES and NMES-based combined interventions can improve lower limb function in stroke patients to varying degrees. Further evidence suggests that, compared with NMES alone, combined NMES interventions may offer additional benefits for functional recovery. For example, the systematic review by Chen et al. [8] reported that NMES significantly improved walking ability in stroke patients, with treatment effects varying across different stages of the disease course. Similarly, Hong et al. [7] confirmed that NMES combined with other rehabilitation interventions significantly improved balance ability, walking ability, and lower limb functional mobility in stroke patients. The findings of the present NMA are generally consistent with these previous results, supporting the positive effects of NMES and NMES-based combined interventions on lower limb dysfunction after stroke. More recently, Liu et al. [48] and He et al. [49] conducted NMA focusing on lower limb dysfunction after stroke. Liu et al. systematically compared the efficacy of different types of electrical stimulation interventions, while He et al. primarily investigated the effects of electrical stimulation on gait function in stroke patients with foot drop, providing important comparative evidence for the application of electrical stimulation in stroke rehabilitation. However, these studies mainly approached the topic from the perspective of stimulation modality or specific clinical conditions, with relatively limited attention to the common clinical practice of combining NMES with different rehabilitation training strategies. Therefore, in contrast to previous studies that mainly relied on traditional pairwise meta-analyses comparing NMES with non-NMES interventions, or network meta-analyses focusing on stimulation types, the present study further compared the relative efficacy of multiple NMES-based combined interventions using an NMA approach. Moreover, we systematically evaluated their potential differences across different functional outcomes (balance ability, walking ability, and lower limb functional mobility) and across different stages of stroke. Notably, although GT+NMES demonstrated overall favorable performance across multiple lower limb functional outcomes, the optimal ranking of NMES-based combined interventions was not consistent across all functional domains. This finding suggests that, in clinical practice, the selection of NMES-based combined interventions may need to be individualized according to specific rehabilitation goals and the stage of stroke in order to achieve optimal rehabilitation outcomes.

After stroke onset, lower limb dysfunction is primarily attributable to damage to the corticospinal tract and an imbalance in motor cortex excitability between the bilateral cerebral hemispheres [5, 50, 51]. NMES delivers regular electrical stimulation to peripheral nerves and muscles, thereby providing the central nervous system with abundant, repetitive, and predictable sensory input and playing an important role in neural network reorganization following stroke. Previous studies have shown that NMES can significantly enhance afferent sensory signals originating from muscle spindles and cutaneous mechanoreceptors. These afferent inputs ascend through spinal pathways and project to the primary somatosensory cortex (S1) and the primary motor cortex (M1), leading to increased neural excitability in the corresponding cortical regions [52]. Such enhanced sensory input is considered a critical prerequisite for inducing post-stroke cortical plasticity, as it facilitates reorganization and compensatory activation of impaired motor cortical areas [53]. In addition, the repetitive sensorimotor stimulation induced by NMES can evoke neuromodulatory effects analogous to Hebbian learning mechanisms, whereby “neurons that fire together are more likely to form stable connections.” This mechanism helps strengthen synaptic transmission efficiency within residual neural pathways and promotes functional reconstruction of cortical networks, thereby providing a central neural basis for motor recovery [54, 55]. Furthermore, neuroimaging and neurophysiological studies have demonstrated that NMES interventions are associated with an expanded extent of activation in motor-related cortical regions and enhanced cortical excitability, changes that are closely correlated with improvements in motor function in stroke patients [56].

Lower limb dysfunction in stroke patients may also be closely associated with structural and functional degeneration of peripheral skeletal muscles. Stroke survivors commonly exhibit muscle atrophy, reduced muscle strength, and impaired muscular endurance in the affected lower limb, all of which directly limit motor output and hinder functional recovery [57, 58]. By repeatedly eliciting contractions of target muscle groups, NMES can induce peripheral adaptations that partially resemble the effects of resistance training, thereby improving muscle morphology and functional properties [6]. Evidence suggests that NMES can increase muscle cross-sectional area, which may attenuate or even reverse post-stroke muscle atrophy resulting from denervation and reduced physical activity, thus providing a structural basis for strength recovery [59]. In terms of muscle strength, the repetitive contractions evoked by NMES can enhance the contractile capacity of muscle fibers and increase force output. Because NMES is capable of recruiting high-threshold motor units that are typically difficult to activate voluntarily in stroke patients due to impaired neural drive, its training effects can directly compensate for deficits in voluntary force production, leading to improvements in both maximal and submaximal muscle strength [60, 61]. In addition, NMES may improve muscular endurance by modulating the metabolic characteristics of muscle fibers. Previous studies have reported that NMES applied over a defined intervention period can increase the activity of oxidative metabolism–related enzymes and alter muscle fiber type composition, thereby enhancing fatigue resistance and the capacity for sustained muscle work [62]. Taken together, these peripheral muscle adaptations provide an important biomechanical foundation for lower limb functional activities such as walking and standing in stroke patients.

Following stroke onset, impairments in proprioceptive input and reduced sensorimotor integration capacity may also contribute substantially to lower limb dysfunction. Proprioceptive deficits can lead to inaccurate central perception of limb position, movement direction, and movement amplitude, thereby compromising the fine control of motor execution [63–65]. By eliciting muscle contractions and activating associated muscle spindles and Golgi tendon organs, NMES provides the central nervous system with abundant, synchronous, and repetitive afferent sensory inputs, thereby enhancing the overall intensity of sensory feedback [66, 67]. This augmented sensory input may facilitate the restoration of functional connectivity within post-stroke sensorimotor circuits, promote the integration of sensory information by the motor cortex, and ultimately improve the accuracy and stability of voluntary motor control. Evidence from two previous meta-analyses has demonstrated that interventions targeting proprioceptive function are significantly associated with improvements in balance performance, walking ability, and lower limb functional mobility in stroke patients [68, 69]. Taken together, these findings suggest that NMES contributes to lower limb functional recovery by enhancing proprioceptive input and optimizing sensorimotor integration processes, thereby providing an important neurophysiological basis for post-stroke rehabilitation.

In this NMA, NMES combined with other rehabilitation interventions generally demonstrated superior effects compared with NMES alone or conventional rehabilitation, suggesting the presence of potential synergistic mechanisms between different interventions. NMES primarily exerts its therapeutic effects by enhancing sensory input, facilitating neuroplasticity and cortical reorganization, and improving peripheral muscle properties, whereas active exercise training or task-oriented rehabilitation mainly focuses on strengthening goal-directed motor output and motor control. These two approaches may therefore complement each other at different levels of the motor control system, forming an important neurophysiological basis for the enhanced efficacy observed with combined interventions. In the present study, CST+NMES ranked highest for balance recovery in stroke patients. This finding is consistent with evidence from a previous systematic review and network meta-analysis, which also reported significant benefits of CST+NMES on post-stroke balance performance [70]. Core stability training primarily enhances control of the trunk and proximal muscle groups, thereby improving postural stability and providing a stable mechanical foundation for lower limb movements [70–72]. NMES may further support postural control by augmenting lower limb sensory afferent input and modulating sensorimotor integration processes. Together, CST and NMES appear to form a complementary interaction at the level of “proximal stability–distal control,” which may contribute to improvements in both static and dynamic postural stability and explain the superior performance of CST+NMES in balance-related outcomes. The present NMA also found that CE+NMES received the highest ranking probability for walking ability in stroke patients, although no comparisons reached statistical significance for this outcome. Previous studies have shown that cycling exercise, as a rhythmic and symmetrical form of lower limb movement, can improve muscle strength and endurance through repeated activation of flexor and extensor muscle groups and facilitate the reconstruction of gait-related motor patterns [73, 74]. By synchronously eliciting muscle contractions and enhancing sensory input within sensorimotor circuits, NMES may improve the consistency and stability of motor output during lower limb movement execution. These two interventions may therefore act synergistically at the level of “motor pattern stabilization–sensory feedback enhancement,” leading to more effective improvements in walking performance after stroke [74, 75]. In addition, based on the network estimates and ranking probabilities, ART+GT+NMES ranked highly for improving lower-limb functional mobility. Moreover, GT+NMES showed relatively favorable performance across multiple domains of lower-limb function. As a highly task-specific rehabilitation approach, gait training promotes recovery of lower limb motor control and overall functional performance by repeatedly practicing movement patterns closely related to real-world walking tasks [76]. NMES may amplify the training effects of gait training by enhancing sensory input and reinforcing sensorimotor integration, thereby contributing to the favorable performance of GT+NMES across multiple lower limb outcomes. Furthermore, the incorporation of ART may provide enriched visual feedback and contextualized movement information, increasing patient engagement and the quality of task execution during training. This may facilitate the learning and transfer of more complex functional activities, which could explain the additional advantages observed with ART+GT+NMES in lower limb functional mobility outcomes [77, 78]. Overall, the differential advantages observed among various NMES-based combined interventions across different outcome measures may reflect differences in task specificity, levels of motor control engagement, and the extent of functional transfer associated with each training modality. Balance ability, walking ability, and lower limb functional mobility represent distinct functional levels ranging from basic postural control to rhythmic movement and further to complex functional tasks. Correspondingly, specific training approaches may exert more targeted effects on their respective functional domains. Within combined interventions, NMES may primarily serve as a modulatory amplifier of training effects rather than the primary determinant of the direction of functional recovery.

The subgroup analyses of this study suggest that the therapeutic effects of different NMES-based combined interventions may vary across stroke patients at different stages of onset. The acute, subacute, and chronic phases of stroke differ substantially in terms of neuroplasticity status, baseline motor function, and rehabilitation goals, which may lead to stage-specific responses to the same intervention [79]. Patients in the acute phase of stroke are typically characterized by impaired postural control, insufficient proximal stability, and markedly reduced overall motor capacity, while also requiring higher levels of safety and tolerance to fatigue during rehabilitation [80]. Rehabilitation during this phase therefore places greater emphasis on establishing basic postural control and proximal stability [81]. In this context, trunk-focused training approaches are generally more acceptable and feasible for patients and can provide a foundational basis for subsequent lower limb functional rehabilitation. Accordingly, CST+NMES may demonstrate particularly favorable effects in acute-stage stroke rehabilitation. In the subacute phase, stroke patients usually experience gradual recovery of voluntary motor function and are better able to participate in higher-intensity and more task-specific training programs. Moreover, functional recovery potential is relatively high during this stage [5]. Rehabilitation priorities progressively shift from basic postural and motor control toward the reconstruction of functional movement patterns, with particular emphasis on lower limb coordination and rhythmic motor control closely related to activities of daily living [82]. Consequently, training approaches that emphasize repeated practice of real-world walking tasks are more likely to yield improvements in walking ability and functional transfer during the subacute phase. GT+NMES may be a promising NMES-based combined intervention for improving lower-limb functional outcomes in patients with subacute stroke. In contrast, functional recovery in the chronic phase relies more heavily on motor learning processes induced by high-repetition, long-term training and multimodal feedback [83, 84]. Single-modality training stimuli are often insufficient to elicit substantial functional improvements at this stage. Instead, comprehensive interventions that integrate multiple training components and enhance patient engagement and training quality may be more effective in promoting further improvements in functional mobility. Accordingly, ART+GT+NMES received favorable ranking probabilities for lower limb functional recovery in patients with chronic stroke, although these subgroup findings require confirmation by future trials with larger sample sizes.

Interpretation of the present findings should take into account several methodological limitations. Some subgroup comparisons were informed by a relatively limited body of evidence, which may reduce the precision of the corresponding effect estimates and increase statistical uncertainty. In addition, because the included studies primarily involved rehabilitation-based interventions, blinding of participants and intervention providers was generally not feasible, as is common in this field. This limitation may have introduced performance-related bias, particularly for outcomes relying on subjective assessment. Furthermore, although random-effects models were employed to account for between-study variability, clinical and methodological diversity among the included trials may have contributed to residual heterogeneity. Potential sources include differences in NMES stimulation parameters (frequency ranging from 20 to 80 Hz), intervention duration (2 to 10 weeks), treatment frequency, time since stroke onset, patient baseline functional status, and variations in the specific protocols used for the co-interventions combined with NMES. These factors may have influenced the magnitude of treatment effects and should be considered when interpreting the pooled estimates. In particular, NMES stimulation parameters such as frequency, pulse width, and session structure may act as effect modifiers that influence treatment outcomes. Meta-regression would be the preferred approach to formally examine the impact of these parameters on effect estimates. However, this analysis was not feasible in the current study for two reasons. First, the number of studies within each direct comparison was generally small, which would have resulted in insufficient statistical power for regression-based analyses. Second, several included studies did not report complete NMES parameter details, further limiting the availability of data for such modeling. Future trials with standardized and fully reported stimulation protocols are needed to enable formal investigation of parameter-outcome relationships through meta-regression. The incomplete reporting of NMES parameters also limits the ability to fully verify the transitivity assumption underlying indirect comparisons in this network. Similarly, conventional rehabilitation, which served as the common comparator and background therapy across most included trials, likely varied in content, dose, and therapist contact time among studies. Because most trials described the control intervention in general terms (e.g., “routine physical therapy” or “standard rehabilitation care”) without detailed specification of treatment components or dosage, it was not feasible to conduct sensitivity analyses stratified by background therapy intensity. This variability in the common comparator may have introduced additional heterogeneity into the indirect comparisons and should be considered when interpreting the relative effect estimates. Moreover, the predominantly star-shaped network geometry meant that the relative rankings among active NMES-based interventions were largely informed by indirect rather than direct evidence. Indirect comparisons inherently carry greater uncertainty than head-to-head trials, and accordingly, the treatment rankings derived from this NMA should be regarded as hypothesis-generating rather than definitive. A sensitivity analysis excluding studies rated as high overall risk of bias was performed to evaluate the robustness of the main findings. Three studies were identified as high risk and removed from the relevant networks. For balance ability and walking ability, the SUCRA ranking orders remained fully consistent with the main analysis. For lower limb functional mobility, the exclusion of high-risk studies led to the loss of the ART+GT+NMES node, while the SUCRA value of GT+NMES increased substantially (from 62.22% to 80.52%), suggesting that the efficacy of GT+NMES may have been underestimated in the main analysis due to the influence of high-risk studies. Overall, the sensitivity analysis supports the robustness of the principal conclusions. The CINeMA assessment further contextualized these findings. For balance ability and lower limb functional mobility, the majority of comparisons were rated as moderate confidence, primarily limited by heterogeneity and imprecision. For walking ability, 90% of comparisons were rated as low confidence, predominantly due to concerns about incoherence arising from the absence of closed loops. These confidence ratings reinforce the need for cautious interpretation of the treatment rankings, particularly for walking ability, and highlight the importance of future head-to-head trials to strengthen the evidence base for NMES-based combined interventions. Because different outcome measures within the same functional domain (e.g., FMA-LE, TUGT, and BS-LE for lower limb functional mobility) were pooled using standardized mean differences, the results reflect relative rather than absolute treatment effects. While this approach is methodologically appropriate for combining studies using heterogeneous measurement scales, it may limit the direct clinical interpretability of the effect estimates. Clinicians should therefore consider the pooled rankings and relative differences in conjunction with scale-specific evidence when making treatment decisions. The restriction to English-language publications may have introduced a language bias. A substantial body of stroke rehabilitation and NMES-related research has been published in non-English languages, particularly in Chinese. Previous methodological studies have suggested that trials published in certain non-English languages may tend to report larger positive effect sizes compared with those published in English. Consequently, the exclusion of non-English studies in this NMA may have led to more conservative effect estimates. Future reviews incorporating multilingual literature searches would help to determine whether the current findings are generalizable beyond the English-language evidence base.

## Conclusion

This study systematically compared the efficacy of different NMES-based combined interventions for improving lower limb dysfunction in stroke patients through NMA. The results indicate that NMES combined with other rehabilitation training is generally superior to NMES alone or conventional rehabilitation, suggesting a potential synergistic effect between NMES and active rehabilitation training. Among the interventions, GT+NMES demonstrated relatively stable ranking performance across multiple lower limb functional outcomes, suggesting it warrants prioritized investigation in future clinical trials. Furthermore, subgroup analyses suggest that stroke patients at different stages of onset respond differently to NMES-based combined interventions, underscoring the importance of stage-specific and individualized rehabilitation strategies.

## Supplementary Information

Below is the link to the electronic supplementary material.

Supplementary Figure 1

Supplementary Figure 2

Supplementary Figure 3

Supplementary Figure 4

Supplementary Figure 5

Supplementary Figure 6

Supplementary Figure 7

Supplementary Figure 8

Supplementary Figure 9

Supplementary Figure 10

Supplementary Figure 11

Supplementary Figure 12

Supplementary Table 1

Supplementary Table 2

Supplementary Table 3

Supplementary Table 4

Supplementary Table 5

Supplementary Table 6

Supplementary Table 7

Supplementary Table 8

Supplementary Table 9

Supplementary Table 10

Supplementary Table 11

Supplementary Table 12

Supplementary Table 13

Supplementary Table 14

Supplementary Table 15

Supplementary Table X

Supplementary Table Y

## Data Availability

The datasets and statistical code used for the network meta-analysis are available from the corresponding author upon reasonable request.

## Abbreviations

NMES: Neuromuscular electrical stimulation
RCTs: Randomized controlled trials
NMA: Network meta-analysis
ROB 2.0: Cochrane Risk of Bias 2.0
PRISMA-NMA: Preferred Reporting Items for Systematic Reviews and Meta-Analyses- Network Meta-Analysis
PROSPERO: International Prospective Register of Systematic Reviews
FES: Functional electrical stimulation
BBS: Berg Balance Scale
PASS: Postural Assessment Scale for Stroke Patients
BS-LE: Brunnstrom Stages–Lower Extremity
FAC: Functional Ambulation Category Scale
10MWT: 10-Meter Walking Test
TUGT: Timed Up and Go Test
FMA-LE: Fugl-Meyer Assessment–Lower Extremity
SMD: Standardized mean differences
CrI: Credible intervals
DIC: Deviance information criterion
SUCRA: Surface under the cumulative ranking curve
C: Conventional rehabilitation
GT: Gait training
CE: Cycling exercise
ART: Augmented reality technology
CST: Core stability training
BT: Balance training
MT: Mirror therapy
MIT: Motor imagery training
PNF: Proprioceptive neuromuscular facilitation
