# Supplementary material for: Comparative effectiveness of neuromuscular electrical stimulation-based combination interventions for post-stroke lower limb rehabilitation: a systematic review and network meta-analysis
Source: J Neuroeng Rehabil. 2026 May 16;23:245. doi: 10.1186/s12984-026-02009-3 (PMC13501765; doi:10.1186/s12984-026-02009-3)
Supplement: Supplementary file 1 [file 12984_2026_2009_MOESM13_ESM.docx]

**1.PubMed**

| **Search number** | **Query** | **Results** |
| --- | --- | --- |
| #1 | "Electric Stimulation Therapy"[Mesh] |  |
| #2 | "Electrotherapy"[Title/Abstract] OR "Therapeutic Electric* Stimulation"[Title/Abstract] OR "Electrical Stimulation Therapy"[Title/Abstract] OR "Interferential Current Electrotherapy"[Title/Abstract] OR "neuromuscular electric* stimulation"[Title/Abstract] OR "neuromuscular electricostimulation"[Title/Abstract] OR "NMES"[Title/Abstract] |  |
| #3 | #1 OR #2 |  |
| #4 | "Stroke"[Mesh] |  |
| #5 | "Stroke*"[Title/Abstract] OR "Cerebrovascular Accident*"[Title/Abstract] OR "CVA*"[Title/Abstract] OR "Brain Vascular Accident*"[Title/Abstract] OR "Apoplex*"[Title/Abstract] OR "acute cerebrovascular lesion"[Title/Abstract] OR "acute focal cerebral vasculopathy"[Title/Abstract] OR "brain accident"[Title/Abstract] OR "brain attack"[Title/Abstract] OR "brain blood flow disturbance"[Title/Abstract] OR "brain insult*"[Title/Abstract] OR "cerebral insult"[Title/Abstract] OR "cerebrovascular arrest"[Title/Abstract] OR "cerebrovascular failure"[Title/Abstract] OR "cerebrovascular injury"[Title/Abstract] OR "cerebrovascular insufficiency"[Title/Abstract] OR "cerebrovascular insult"[Title/Abstract] OR "cerebrum vascular accident"[Title/Abstract] OR "insultus cerebralis"[Title/Abstract] OR "ischaemic seizure"[Title/Abstract] OR "ischemic seizure"[Title/Abstract] |  |
| #6 | #4 OR #5 |  |
| #7 | "Random*"[Title/Abstract] |  |
| #8 | #3 AND #6 AND #7 | 1563 |

**2.Cochrane Library**

| **Search number** | **Query** | **Results** |
| --- | --- | --- |
| #1 | MeSH descriptor: [Electric Stimulation Therapy] explode all trees |  |
| #2 | (Electrotherapy):ti,ab,kw OR (Therapeutic Electric* Stimulation):ti,ab,kw OR (Electrical Stimulation Therapy):ti,ab,kw OR (Interferential Current Electrotherapy):ti,ab,kw OR (neuromuscular electric* stimulation):ti,ab,kw |  |
| #3 | (neuromuscular electricostimulation):ti,ab,kw OR (NMES):ti,ab,kw |  |
| #4 | #1 OR #2 OR #3 |  |
| #5 | MeSH descriptor: [Stroke] explode all trees |  |
| #6 | (Stroke*):ti,ab,kw OR (Cerebrovascular Accident*):ti,ab,kw OR (CVA*):ti,ab,kw OR (Brain Vascular Accident*):ti,ab,kw OR (Apoplex*):ti,ab,kw |  |
| #7 | (acute cerebrovascular lesion):ti,ab,kw OR (acute focal cerebral vasculopathy):ti,ab,kw OR (brain accident):ti,ab,kw OR (brain attack):ti,ab,kw OR (brain blood flow disturbance):ti,ab,kw |  |
| #8 | (brain insult*):ti,ab,kw OR (cerebral insult):ti,ab,kw OR (cerebrovascular arrest):ti,ab,kw OR (cerebrovascular failure):ti,ab,kw OR (cerebrovascular injury):ti,ab,kw |  |
| #9 | (cerebrovascular insufficiency):ti,ab,kw OR (cerebrovascular insult):ti,ab,kw OR (cerebrum vascular accident):ti,ab,kw OR (insultus cerebralis):ti,ab,kw OR (ischaemic seizure):ti,ab,kw |  |
| #10 | (ischemic seizure):ti,ab,kw |  |
| #11 | #5 OR #6 OR #7 OR #8 OR #9 OR #10 |  |
| #12 | (Random*):ti,ab,kw |  |
| #13 | #4 OR #11 OR #12 | 1886 |

**3.Embase**

| **Search number** | **Query** | **Results** |
| --- | --- | --- |
| #1 | 'neuromuscular electrical stimulation'/exp |  |
| #2 | Electrotherapy:ab,ti OR Therapeutic Electric* Stimulation:ab,ti OR Electrical Stimulation Therapy:ab,ti OR Interferential Current Electrotherapy:ab,ti OR neuromuscular electric* stimulation:ab,ti OR neuromuscular electricostimulation:ab,ti OR NMES:ab,ti |  |
| #3 | #1 OR #2 |  |
| #4 | 'cerebrovascular accident'/exp |  |
| #5 | Stroke*:ab,ti OR Cerebrovascular Accident*:ab,ti OR CVA*:ab,ti OR Brain Vascular Accident*:ab,ti OR Apoplex*:ab,ti OR acute cerebrovascular lesion:ab,ti OR acute focal cerebral vasculopathy:ab,ti OR brain accident:ab,ti OR brain attack:ab,ti OR brain blood flow disturbance:ab,ti OR brain insult*:ab,ti OR cerebral insult:ab,ti OR cerebrovascular arrest:ab,ti OR cerebrovascular failure:ab,ti OR cerebrovascular injury:ab,ti OR cerebrovascular insufficiency:ab,ti OR cerebrovascular insult:ab,ti OR cerebrum vascular accident:ab,ti OR insultus cerebralis:ab,ti OR ischaemic seizure:ab,ti OR ischemic seizure:ab,ti |  |
| #6 | #4 OR #5 |  |
| #7 | Random*:ab,ti |  |
| #8 | #3 AND #6 AND #7 | 405 |

**4.Web of science**

| **Search number** | **Query** | **Results** |
| --- | --- | --- |
| #1 | Electrotherapy (Topic) OR Therapeutic Electric* Stimulation (Topic) OR Electrical Stimulation Therapy (Topic) OR Interferential Current Electrotherapy (Topic) OR neuromuscular electric* stimulation (Topic) OR neuromuscular electricostimulation (Topic) OR NMES (Topic) |  |
| #2 | Stroke* (Topic) OR Cerebrovascular Accident* (Topic) OR CVA* (Topic) OR Brain Vascular Accident* (Topic) OR Apoplex* (Topic) OR acute cerebrovascular lesion (Topic) OR acute focal cerebral vasculopathy (Topic) OR brain accident (Topic) OR brain attack (Topic) OR brain blood flow disturbance (Topic) OR brain insult* (Topic) OR cerebral insult (Topic) OR cerebrovascular arrest (Topic) OR cerebrovascular failure (Topic) OR cerebrovascular injury (Topic) OR cerebrovascular insufficiency (Topic) OR cerebrovascular insult (Topic) OR cerebrum vascular accident (Topic) OR insultus cerebralis (Topic) OR ischaemic seizure (Topic) OR ischemic seizure (Topic) |  |
| #3 | Random* (Topic) |  |
| #4 | #1 AND #2 AND #3 | 938 |
