# Supplementary material for: Comparative effectiveness of neuromuscular electrical stimulation-based combination interventions for post-stroke lower limb rehabilitation: a systematic review and network meta-analysis
Source: J Neuroeng Rehabil. 2026 May 16;23:245. doi: 10.1186/s12984-026-02009-3 (PMC13501765; doi:10.1186/s12984-026-02009-3)
Supplement: Supplementary file 2 [file 12984_2026_2009_MOESM14_ESM.docx]

**Supplementary Table 2 league table for balance ability in acute stroke patients.**

| **Balance Ability** | | | |
| --- | --- | --- | --- |
| **C** |  |  |  |
| -1.07 (-1.95, -0.19) | **CST+NMES** |  |  |
| -0.11 (-1.49, 1.25) | 0.95 (-0.67, 2.57) | **GT+NMES** |  |
| -0.72 (-1.36, -0.05) | 0.35 (-0.59, 1.31) | -0.6 (-2.1, 0.92) | **NMES** |

NMES: neuromuscular electrical stimulation; C: conventional rehabilitation; GT: gait training; CST: core stability training.
