# Supplementary material for: Comparative effectiveness of neuromuscular electrical stimulation-based combination interventions for post-stroke lower limb rehabilitation: a systematic review and network meta-analysis
Source: J Neuroeng Rehabil. 2026 May 16;23:245. doi: 10.1186/s12984-026-02009-3 (PMC13501765; doi:10.1186/s12984-026-02009-3)
Supplement: Supplementary file 3 [file 12984_2026_2009_MOESM15_ESM.docx]

**Supplementary Table 3 SUCRA ranking table for balance ability in acute stroke patients.**

| **Treatment** | **Balance Ability** | **Lower Limb Functional Mobility** |
| --- | --- | --- |
| ART+GT+NMES | NR | NR |
| BT+NMES | NR | NR |
| C | 15.00 | 5.86 |
| CST+NMES | 89.78 | NR |
| GT+NMES | 28.30 | 84.43 |
| MIT+NMES | NR | NR |
| MT+NMES | NR | NR |
| NMES | 66.92 | 59.71 |
| CE+NMES | NR | NR |
| PNF+NMES | NR | NR |

NMES: neuromuscular electrical stimulation; C: conventional rehabilitation; GT: gait training; CE: cycling exercise; ART: augmented reality technology; CST: core stability training; BT: balance training; MT: mirror therapy; MIT: motor imagery training; PNF: proprioceptive neuromuscular facilitation; NR: no report.
