# Supplementary material for: Comparative effectiveness of neuromuscular electrical stimulation-based combination interventions for post-stroke lower limb rehabilitation: a systematic review and network meta-analysis
Source: J Neuroeng Rehabil. 2026 May 16;23:245. doi: 10.1186/s12984-026-02009-3 (PMC13501765; doi:10.1186/s12984-026-02009-3)
Supplement: Supplementary file 4 [file 12984_2026_2009_MOESM16_ESM.docx]

**Supplementary Table 4 league table for lower limb functional mobility in acute stroke patients.**

| **Lower Limb Functional Mobility** | | |
| --- | --- | --- |
| **C** |  |  |
| -1.07 (-2.6, 0.45) | **GT+NMES** |  |
| -0.51 (-1.11, 0.09) | 0.56 (-1.08, 2.19) | **NMES** |

NMES: neuromuscular electrical stimulation; C: conventional rehabilitation; GT: gait training.
