# Supplementary material for: Comparative effectiveness of neuromuscular electrical stimulation-based combination interventions for post-stroke lower limb rehabilitation: a systematic review and network meta-analysis
Source: J Neuroeng Rehabil. 2026 May 16;23:245. doi: 10.1186/s12984-026-02009-3 (PMC13501765; doi:10.1186/s12984-026-02009-3)
Supplement: Supplementary file 5 [file 12984_2026_2009_MOESM17_ESM.docx]

**Supplementary Table 5 league table for balance ability in subacute stroke patients.**

| **Balance Ability** | | | |
| --- | --- | --- | --- |
| **BT+NMES** |  |  |  |
| -3.11 (-20.85, 14.56) | **C** |  |  |
| -8.04 (-32.35, 16.3) | -4.91 (-21.52, 11.63) | **GT+NMES** |  |
| -16.06 (-42.14, 10.12) | -12.93 (-32.09, 6.23) | -8.01 (-33.25, 17.19) | **NMES** |

NMES: neuromuscular electrical stimulation; C: conventional rehabilitation; GT: gait training; BT: balance training.
