# Supplementary material for: Comparative effectiveness of neuromuscular electrical stimulation-based combination interventions for post-stroke lower limb rehabilitation: a systematic review and network meta-analysis
Source: J Neuroeng Rehabil. 2026 May 16;23:245. doi: 10.1186/s12984-026-02009-3 (PMC13501765; doi:10.1186/s12984-026-02009-3)
Supplement: Supplementary file 7 [file 12984_2026_2009_MOESM19_ESM.docx]

**Supplementary Table 7 league table for walking ability in subacute stroke patients.**

| **Walking Ability** | | | |
| --- | --- | --- | --- |
| **C** |  |  |  |
| -0.65 (-1.55, 0.23) | **CE+NMES** |  |  |
| -0.01 (-1.14, 1.14) | 0.65 (-0.78, 2.11) | **GT+NMES** |  |
| -0.37 (-0.86, 0.01) | 0.28 (-0.75, 1.23) | -0.38 (-1.62, 0.81) | **NMES** |

NMES: neuromuscular electrical stimulation; C: conventional rehabilitation; GT: gait training; CE: cycling exercise.
