# Supplementary material for: Comparative effectiveness of neuromuscular electrical stimulation-based combination interventions for post-stroke lower limb rehabilitation: a systematic review and network meta-analysis
Source: J Neuroeng Rehabil. 2026 May 16;23:245. doi: 10.1186/s12984-026-02009-3 (PMC13501765; doi:10.1186/s12984-026-02009-3)
Supplement: Supplementary file 8 [file 12984_2026_2009_MOESM20_ESM.docx]

**Supplementary Table 8 league table for lower limb functional mobility in subacute stroke patients**

| **Lower Limb Functional Mobility** | | | | |
| --- | --- | --- | --- | --- |
| **C** |  |  |  |  |
| -1.6 (-3.3, 0.08) | **GT+NMES** |  |  |  |
| -0.7 (-2.41, 1.03) | 0.9 (-1.5, 3.32) | **MIT+NMES** |  |  |
| -1.63 (-3.26, 0) | -0.04 (-2.38, 2.31) | -0.93 (-3.29, 1.44) | **MT+NMES** |  |
| -0.41 (-1.45, 0.42) | 1.18 (-0.84, 3.02) | 0.28 (-1.77, 2.14) | 1.22 (-0.76, 3.01) | **NMES** |

NMES: neuromuscular electrical stimulation; C: conventional rehabilitation; GT: gait training; MT: mirror therapy; MIT: motor imagery training.
