# Supplementary material for: Comparative effectiveness of neuromuscular electrical stimulation-based combination interventions for post-stroke lower limb rehabilitation: a systematic review and network meta-analysis
Source: J Neuroeng Rehabil. 2026 May 16;23:245. doi: 10.1186/s12984-026-02009-3 (PMC13501765; doi:10.1186/s12984-026-02009-3)
Supplement: Supplementary file 9 [file 12984_2026_2009_MOESM21_ESM.docx]

**Supplementary Table 9 league table for balance ability in chronic stroke patients**

| **Balance Ability** | | | | | | |
| --- | --- | --- | --- | --- | --- | --- |
| **ART+GT+NMES** |  |  |  |  |  |  |
| 1.07 (-6.02, 8.49) | **BT+NMES** |  |  |  |  |  |
| 2.01 (-2.74, 7.05) | 0.93 (-4.37, 6.23) | **C** |  |  |  |  |
| -1.67 (-8.88, 5.83) | -2.76 (-10.3, 4.8) | -3.7 (-9.12, 1.77) | **CST+NMES** |  |  |  |
| 0.29 (-4.75, 5.05) | -0.74 (-7.02, 4.82) | -1.7 (-4.64, 0.66) | 1.99 (-4.4, 7.76) | **GT+NMES** |  |  |
| -5.59 (-15.71, 4.49) | -6.71 (-17.11, 3.44) | -7.63 (-16.66, 1.21) | -3.96 (-14.44, 6.2) | -5.84 (-15.16, 3.43) | **MT+NMES** |  |
| 1.47 (-7.39, 10.52) | 0.36 (-8.75, 9.53) | -0.57 (-8.2, 7.06) | 3.11 (-6.07, 12.36) | 1.21 (-6.66, 9.41) | 7.06 (-4.58, 18.87) | **PNF+NMES** |

NMES: neuromuscular electrical stimulation; C: conventional rehabilitation; GT: gait training; CST: core stability training; BT: balance training; MT: mirror therapy; PNF: proprioceptive neuromuscular facilitation; ART: augmented reality technology.
