# Supplementary material for: Comparative effectiveness of neuromuscular electrical stimulation-based combination interventions for post-stroke lower limb rehabilitation: a systematic review and network meta-analysis
Source: J Neuroeng Rehabil. 2026 May 16;23:245. doi: 10.1186/s12984-026-02009-3 (PMC13501765; doi:10.1186/s12984-026-02009-3)
Supplement: Supplementary file 10 [file 12984_2026_2009_MOESM22_ESM.docx]

**Supplementary Table 10 SUCRA ranking for balance ability in chronic stroke patients.**

| **Treatment** | **Balance Ability** | **Walking Ability** | **Lower Limb Functional Mobility** |
| --- | --- | --- | --- |
| ART+GT+NMES | 52.28 | NR | 77.00 |
| BT+NMES | 35.47 | NR | 49.84 |
| C | 16.38 | 51.91 | 21.40 |
| CST+NMES | 72.34 | 54.21 | 45.19 |
| GT+NMES | 49.57 | 9.81 | 60.57 |
| MIT+NMES | NR | NR | NR |
| MT+NMES | 88.91 | NR | 36.11 |
| NMES | NR | 84.07 | 59.88 |
| CE+NMES | NR | NR | NR |
| PNF+NMES | 34.74 | NR | NR |

NMES: neuromuscular electrical stimulation; C: conventional rehabilitation; GT: gait training; CE: cycling exercise; ART: augmented reality technology; CST: core stability training; BT: balance training; MT: mirror therapy; MIT: motor imagery training; PNF: proprioceptive neuromuscular facilitation; NR: no report.
