# Supplementary material for: Comparative effectiveness of neuromuscular electrical stimulation-based combination interventions for post-stroke lower limb rehabilitation: a systematic review and network meta-analysis
Source: J Neuroeng Rehabil. 2026 May 16;23:245. doi: 10.1186/s12984-026-02009-3 (PMC13501765; doi:10.1186/s12984-026-02009-3)
Supplement: Supplementary file 11 [file 12984_2026_2009_MOESM23_ESM.docx]

**Supplementary Table 11 league table for walking ability in chronic stroke patients.**

| **Walking Ability** | | | |
| --- | --- | --- | --- |
| **C** |  |  |  |
| -0.03 (-0.66, 0.6) | **CST+NMES** |  |  |
| 0.31 (-0.14, 0.7) | 0.34 (-0.45, 1.06) | **GT+NMES** |  |
| -0.3 (-0.93, 0.33) | -0.27 (-1.15, 0.61) | -0.61 (-1.35, 0.17) | **NMES** |

NMES: neuromuscular electrical stimulation; C: conventional rehabilitation; GT: gait training; CST: core stability training.
