# Supplementary material for: Comparative effectiveness of neuromuscular electrical stimulation-based combination interventions for post-stroke lower limb rehabilitation: a systematic review and network meta-analysis
Source: J Neuroeng Rehabil. 2026 May 16;23:245. doi: 10.1186/s12984-026-02009-3 (PMC13501765; doi:10.1186/s12984-026-02009-3)
Supplement: Supplementary file 12 [file 12984_2026_2009_MOESM24_ESM.docx]

**Supplementary Table 12 league table for lower limb functional mobility in chronic stroke patients**

| **Lower Limb Functional Mobility** | | | | | | |
| --- | --- | --- | --- | --- | --- | --- |
| **ART+GT+NMES** |  |  |  |  |  |  |
| 0.77 (-2.29, 3.85) | **BT+NMES** |  |  |  |  |  |
| 1.38 (-0.47, 3.26) | 0.61 (-1.84, 3.05) | **C** |  |  |  |  |
| 0.89 (-2.13, 3.95) | 0.14 (-3.28, 3.52) | -0.48 (-2.87, 1.91) | **CST+NMES** |  |  |  |
| 0.53 (-1.34, 2.37) | -0.23 (-2.91, 2.35) | -0.84 (-1.86, 0.09) | -0.36 (-3, 2.19) | **GT+NMES** |  |  |
| 1.17 (-1.88, 4.29) | 0.42 (-3.07, 3.87) | -0.2 (-2.68, 2.27) | 0.28 (-3.17, 3.71) | 0.64 (-1.96, 3.35) | **MT+NMES** |  |
| 0.46 (-2.59, 3.59) | -0.3 (-3.76, 3.16) | -0.92 (-3.36, 1.54) | -0.43 (-3.86, 2.98) | -0.07 (-2.66, 2.6) | -0.71 (-4.19, 2.77) | **NMES** |

NMES: neuromuscular electrical stimulation; C: conventional rehabilitation; GT: gait training; ART: augmented reality technology; CST: core stability training; BT: balance training; MT: mirror therapy.
