# Supplementary material for: Comparative effectiveness of neuromuscular electrical stimulation-based combination interventions for post-stroke lower limb rehabilitation: a systematic review and network meta-analysis
Source: J Neuroeng Rehabil. 2026 May 16;23:245. doi: 10.1186/s12984-026-02009-3 (PMC13501765; doi:10.1186/s12984-026-02009-3)
Supplement: Supplementary file 13 [file 12984_2026_2009_MOESM29_ESM.docx]

**Supplementary Table Y Number of studies and total participants contributing to each intervention node across outcomes.**

| Intervention | Balance Ability | | Walking Ability | | Lower Limb Functional Mobility | |
| --- | --- | --- | --- | --- | --- | --- |
|  | Studies | Participants | Studies | Participants | Studies | Participants |
| NMES | 6 | 106 | 7 | 190 | 9 | 234 |
| GT+NMES | 8 | 270 | 7 | 304 | 10 | 357 |
| CST+NMES | 3 | 80 | 1 | 60 | 1 | 60 |
| ART+GT+NMES | 1 | 9 | NR | NR | 1 | 9 |
| CE+NMES | NR | NR | 1 | 19 | NR | NR |
| BT+NMES | 2 | 32 | NR | NR | 1 | 25 |
| MT+NMES | 1 | 14 | NR | NR | 2 | 37 |
| MIT+NMES | NR | NR | NR | NR | 1 | 10 |
| PNF+NMES | 1 | 17 | NR | NR | NR | NR |
| C | 20 | 526 | 16 | 595 | 24 | 735 |

Interventions informed by only one or two studies should be interpreted with particular caution due to limited evidence. NR: not reported.
